# Thermodynamic and Atomistic Insights into Lignin Solubility from Experimentally Validated Molecular Dynamics Simulations

**DOI:** 10.1021/acs.biomac.5c02503

**Published:** 2026-04-21

**Authors:** Klara Hackenstrass, Nissa Nurfajrin Solihat, Paula Nousiainen, Monika Österberg, Sara Florisson, Jakob Wohlert, Malin Wohlert

**Affiliations:** † Department of Materials Science and Engineering, 8097 Uppsala University, SE-752 37 Uppsala, Sweden; ‡ Department of Bioproducts and Biosystems, School of Chemical Engineering, 174277 Aalto University, FI-00076 Espoo, Finland; ∥ Department of Fibre and Polymer Technology, KTH Royal Institute of Technology, SE-100 44 Stockholm, Sweden

## Abstract

Lignin solubility is critical for extraction, tailoring processing, and downstream applications. Understanding the factors governing its solubility helps understand the behavior of this chemically heterogeneous aromatic biopolymer that has found increasing use in recent years. This study explores the molecular weight dependence of lignin solubility across different solvents. Molecular dynamics simulations are linked to experimental solubility measurements via a ranking of solvents. This integrated approach combines the strength of atomistic insights from simulations with experimental validation. The free energy of solvation and its enthalpic and entropic contributions are determined, and specific interactions such as hydrogen bonds and π–π stacking are quantified. The results show that differences in lignin solubilization efficiency between organic solvents and water are primarily enthalpy-driven with a smaller entropic contribution. In contrast to simplified solubility parameter approaches, molecular dynamics simulations provide a holistic view of lignin solubility and are shown to be a useful tool for understanding its solubilization mechanisms.

## Graphical abstract

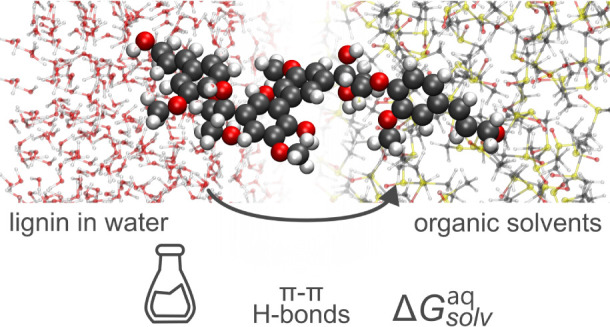

## Introduction

Lignin extraction from biomass, processing, and application are intrinsically connected with the choice of solvent.^1–4^ This choice not only governs the maximum amount of solute that can be dissolved under given conditions, or solubility, but also self-association and interaction with other biopolymers.^5–7^ These solvent-dependent properties are critical in laboratory-scale research of nanoparticle formation via antisolvent techniques or fractionation of industrial lignin samples into more uniform subsets.^8–11^ As such, a comprehensive understanding of lignin solubility and its behavior across various solvents is essential for optimizing extraction and advancing both laboratory-scale research and industrial applications of lignin as a versatile biopolymer.

Lignin solubility is governed by its chemical structure, which is highly heterogeneous. Extracted lignins show large variations in molecular weight and chemical structure, depending on the source and extraction methods.^12,13^ Native lignin is produced in the plant cell wall through radical coupling, showing a large chemical variety of the polymer. Lignin consists of a random sequence of aromatic phenylpropyl units, mainly *p*-hydroxyphenyl (H), guaiacyl (G), and syringyl (S) units, with various interconnecting linkages.^14,15^ The prevalence of each unit and linkage depends on the plant species, as well as the location and tissue within said plant, making characterization difficult.^16,17^ The structural variability of technical lignins arises therefore from its intrinsic heterogeneity and changes made to the structure during chemical and physical processing during extraction, as well as inconsistencies when comparing results from different analytical techniques.^18^ While the exact percentages might vary, it is generally agreed upon that native lignin in softwoods, such as Norway spruce (*Picea abies*), is mainly comprised of G-units (95%) connected by β-O4′ (50%) and 5-5′ (24%) interunit linkages.^15,19,20^ Ether linkages, such as β-O4′ are cleaved first during processing, while the carbon–carbon 5-5′ linkages can withstand harsh chemical environments. This leads to a higher representation of the 5-5′ linkage in industrial lignins.^21,22^

The solubility of various lignin samples has been studied experimentally in water, organic solvents, and ionic liquids.^1,23^ As a compound that describes a wider group of molecules, differences in the solubility between lignin samples are inherently seen. Hydrogen bonding and π–π stacking are often suggested to explain observed differences in solubility between different solvents and lignin from various sources. Hydrogen bonds can occur between the hydroxyl and ether groups in the lignin molecule and with polar solvent molecules, while the hydrophobic aromatic rings in the lignin molecule can interact via π–π stacking.^24^ Hydrogen bonds and π–π stacking can be observed and quantified with density functional theory (DFT) calculations and molecular dynamics (MD) simulations. In DFT calculations, the electronic structure of molecules is determined, showing the preferential sites on lignin fragments for hydrogen bonds with other lignin or solvent molecules and alignment of aromatic rings into π–π stacking.^25–27^ Classical force field MD simulations give the static properties and dynamic evolution of the lignin molecules in solvent, showing that lignin molecules which had stayed dispersed in ethanol formed clusters in water.^6^ High-molecular-weight lignin molecules exhibit different extensions depending on the solvent, solvent mixture ratio, and temperature.^28,29^ Analyzing the radial distribution function across different functional groups showed the importance of lignin’s hydroxyl groups in interactions with the solvent, forming intramolecular hydrogen bonds if not interacting with the solvent.^28^ Recent advances have been made in quantifying the π–π stacking in lignin. The lignin force field is not optimized for this interaction, so the focus is on comparing systems rather than absolute quantification.^30–32^

There are various approaches to describe and predict solubility with varying theoretical backgrounds. In this context, the Hansen Solubility Parameters (HSP) and Kamlet–Taft (KT) parameters are two concepts, each using three parameters to describe the solute–solvent system.^33,34^ The HSP have been calculated for kraft lignin from experiments and are, together with the KT parameters, used in COSMO-RS calculations of infinite dilution activity coefficients.^35,36^ The latter showed that the composition of lignin molecules with the same number of phenylpropyl units can change the solubility in a solvent, describing potential solvents sensitive to lignin composition.^36^ Another quantity related to solubility is the free energy of solvation, Δ*G*_solv_. It describes the difference in Gibbs free energy of a molecule in the solvent and gas phases, which is part of the energy required for the dissolution of a molecule and is unique to the solvent. This property has been calculated for small lignin fragments, showing a lower free energy of solvation for the S monolignol compared to G monolignol.^28,37^ The enthalpic Δ*H* and entropic Δ*S* contributions to Δ*G*_solv_ can be calculated from its temperature dependence, providing further insights and a thermodynamic understanding of solubility. To the authors’ knowledge, this quantification of both the enthalpy and especially entropy has not been done for lignin.

The goal of the present study is to gain a comprehensive understanding of lignin solubility through a simulation-driven approach, complemented by experimental validation. Free energy of solvation calculations and simulations of cluster formation provide insights into molecular interactions at the atomistic scale and the thermodynamics behind lignin solubility. The enthalpic and entropic contributions were calculated through the free energy of solvation simulations using Replica Exchange Molecular Dynamics (REMD). The simulation results were verified against experimentally measured solubility data of both model compounds and industrial lignin samples via solvent ranking. This strategy combines the strength of molecular-level interpretation of lignin–solvent interactions from atomistic-scale simulations with validation and extension to industrial compounds through experiments.

## Methods

The present study consists of three distinct parts: 1) experimental determination of the soluble fraction of various lignin model compounds and industrial lignin samples, 2) classical MD simulations to characterize and compare intermolecular interactions such as hydrogen bonding and π–π stacking between systems, and finally, 3) free energy of solvation simulations to evaluate the free energy of solvation of each compound relative to water and a solid phase (powder) reference, mirroring the experimental measurements of the soluble fraction. An overview of the overall workflow is presented in Figure 1. A shared metric to compare among all methods is a ranking of solvents.

**Figure 1:**
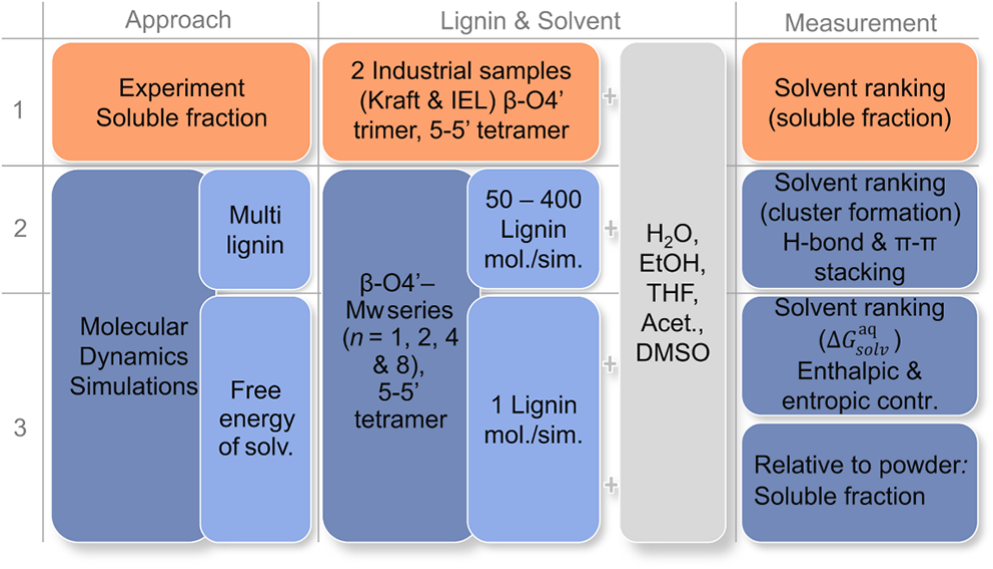
Overview of the experimental and simulation approaches used to evaluate the lignin solubility, lignin-ligninand lignin-solvent interactions.

### Experiments

#### Materials

Two lignin types and two lignin model compounds were used for solubility experiments: industrial softwood kraft lignin BioPiva 395 (UPM, Finland), ethanolic biorefinery-based hardwood poplar lignin (IEL, Italian Bio Products SRL, Crescentino, Italy) and trimeric and tetrameric lignin model compounds. Both industrial samples were free of carbohydrate complexes (LCCs). The IEL lignin was isolated from crude residual lignin by reflux in a hot ethanol fractionation method described by Maltari et al.^38^ The tetrameric β-O4′ biphenyl-type lignin model compound (Figure S1a) was synthesized via oxidative dimerization of the corresponding dimeric β-O4′ lignin model compound using enzymatic catalysis by horseradish peroxidase (HRP) and hydrogen peroxide (H_2_O_2_).^39^ The trimeric guaiacyl β-O4′-type model compound (Figure S1b) was synthesized by a modified multistep synthesis procedure according to Lahtinen et al.^40^ The lignin samples were analyzed by ^31^P nuclear magnetic Resonance (NMR) to quantitatively determine their various hydroxyl groups and by heteronuclear single quantum coherence (HSQC) NMR (Figures S2–S5) to determine their structural interconnecting unit distribution. The structures of the synthesized lignin model compounds and the predicted structures of lignins are shown in Figure S1, and the chemical compositions of industrial lignins are listed in Table S1. Ethanol (EtOH) and tetrahydrofuran (THF) were obtained from Sigma-Aldrich, Germany. Acetone and dimethyl sulfoxide (DMSO) were purchased from Merck, Germany. All solvents were of analytical grade and used without purification.

#### Methods

The adopted lignin solubility protocol followed the previous study by Dastpak et al.^23^ Lignins and lignin model compounds were dissolved at 2 wt % in five solvents: water, ethanol, THF, acetone and DMSO. The solution was stirred at a speed between 300 and 400 rpm at room temperature for 24 h before being centrifuged at 5000 rpm (Eppendorf 5804, Germany) for 10 min. The insoluble fraction in lignin samples was dried overnight at 105 °C, while lignin model compounds were dried for 2 h in an oven under vacuum pressure below 50 mbar at 30 °C. The mean soluble fraction of three replications was determined by deducting the insoluble fraction from the initial weight.

### Simulations

#### Simulation Protocol

All simulations used guaiacyl-type lignin models and focused on two molecular variations: molecular weight and a single linkage modification, reflecting a structural difference between native and kraft lignin. The effect of molecular weight was examined by comparing molecules with varying numbers of phenylpropyl units connected by β-O4′ linkages. These units are also termed rings, given the aromatic ring in each phenylpropyl unit. The molecular weight series of lignin comprises monomer (one phenylpropyl unit or ring), dimer (two rings), tetramer (four rings), and octamer (eight rings) species. An additional tetramer with the linkage sequence β-O4′-5-5′-β-O4′ (referred to as 5-5′) was compared with the aforementioned pure β-O4′ tetramer (referred to as β-O4′). The chemical structures of all molecules can be found in Figure S6.

The simulations were done in Gromacs 2023 to 2025.2.^41^ The lignin models were built using functions from TopoGromacs and TopoTools implemented in VMD.^42–44^ The system was parametrized using the lignin-specific CHARMM36 force field.^45^ The TIP3P model was used for water.^46^ ethanol, THF, acetone, and DMSO were parametrized using the CHARMM general force field (CGenFF).^47^ A validation of this combination of force fields was done by Vermaas et al., showing diffusion coefficients and comparing free energy of solvation values of smaller molecules, similar to lignin fragments, with literature values obtained experimentally and through quantum mechanics/molecular mechanics calculations.^28^

#### Multilignin Simulations

The aim of these simulations is to observe lignin–lignin and lignin–solvent interactions. Each simulation involves one type of lignin and one type of solvent. The number of lignin molecules was adjusted to the size of the molecule: 400 monomers, 200 dimers, 100 tetramers, and 50 octamers, maintaining 400 aromatic rings per simulation. The lignin molecules were randomly placed in the 10 × 10 × 10 nm^3^ simulation box and subsequently dissolved. The same five solvents as in the experiments were used, and solvent molecules were added to achieve the experimental density. The simulations were minimized and equilibrated using a sequence of NVT and NPT calculations with the Berendsen thermostat.^48^ The first 100 ns of the production run were used as additional equilibration, whereas the subsequent 100 ns were used for analysis (Equilibration Figures S7–S9). This part of the simulation is done without replicates and is considered an illustrative way to show the formation of clusters or the lack thereof, as well as to give indications of interactions. All production runs were done with the leapfrog algorithm at 300 K and 1 bar using the c-rescale barostat (τ_P_ = 5 ps) and the Bussi–Donadio–Parrinello thermostat (τ_T_ = 1 ps), respectively.^49,50^ The simulation time step was 2 fs, and all bonds were constrained using P-LINCS.^51^ Lennard-Jones and Coulomb interactions were truncated at 1.2 nm. The particle-mesh Ewald method was used to calculate long-range electrostatic interactions and the dispersion correction for long-range van der Waals interactions.^52,53^

Hydrogen bonds and π–π stacking were identified based on geometric criteria. The GROMACS *hbond* tool was used with a distance cutoff of 0.35 nm and a Hydrogen-Donor–Acceptor angle cutoff of 30° and values recorded every 0.1 ns.^54^ π–π stacking was defined by the distance between the centroids of the rings *d*, the angle between the ring planes α, and the lateral displacement of the rings *l* and was calculated every 1 ns over the production run. The accepted ranges for sandwich stacking are *d* = 0.32–0.46 nm, α = 10–50°, and *l* = 0.00–0.20 nm and T-shaped stacking *d* = 0.40–0.60 nm, α = 70–90°, and *l* = 0.00–0.20 nm.^30^ Error bars indicate the width of the distribution of numbers of hydrogen bonds and π–π stacking and are calculated as standard deviations of the production runs.

#### Free-Energy of Solvation Simulations

Transfer free energies were calculated using the thermodynamic cycle (Figure 2), where $\Delta G_{\mathrm{solv}}^{\mathrm{aq}}$ (i.e. solubility relative to water) was calculated using simulations going from the decoupled state to water $\left(\Delta G_{\mathrm{solv}}^{\mathrm{water}}\right)$ and from the decoupled state to another solvent $\left(\Delta G_{\mathrm{solv}}^{\mathrm{solvent}}\right)$ . For the simulations relative to a system mimicking a powder, the thermodynamic cycle contains a system with a solid lignin particle. In these calculations, a lignin molecule on the surface of a lignin cluster was decoupled from the interactions with the lignin molecules instead of a solvent.

**Figure 2:**
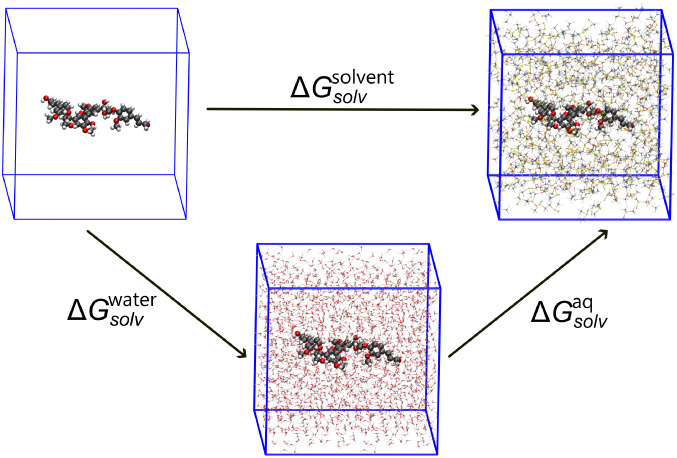
Graphical representation of the thermodynamic cycle used to calculate the transfer free energy $\Delta G_{\mathrm{solv}}^{\mathrm{aq}}$ from decoupling solute in water $\Delta G_{\mathrm{solv}}^{\mathrm{water}}$ and solvent $\Delta G_{\mathrm{solv}}^{\mathrm{solvent}}$ .

The systems were decoupled through a stepwise removal of the Coulomb interactions λ_C_ = (1.0, 0.8, 0.6, 0.4, 0.2, 0.1, 0.0) and subsequently van der Waals interactions λ_vdW_ = (1.0, 0.9, 0.8, 0.6, 0.4, 0.3, 0.25, 0.2, 0.15, 0.1, 0.05, 0.0) between the lignin molecule and the surrounding molecules. The values for λ were determined based on simulations with higher λ resolution, focusing on steps with the largest change, aiming for a compromise between accuracy and computational resources. The values and errors for each step of removing the interactions can be seen in Figure S10 for an exemplary system. Bennett’s acceptance ratio was used to calculate the free energy profile.^55^ For each lambda state, the systems were minimized and equilibrated using a sequence of NVT and NPT calculations using the Berendsen thermostat.^48^ The NPT ensemble was used for the second step of the equilibration and the production, except in the simulations of the lignin particle in a vacuum, which required the NVT ensemble. The production run was done with the leapfrog stochastic dynamics integrator for 5 ns, with an additional 100 ps for equilibration. REMD was performed over the temperature range of 300 to 360 K with the Bussi–Donadio–Parrinello thermostat (τ_T_ = 5 ps).^50^ The numbers of replicas ranged between 9 and 21 and were set to obtain an exchange rate between the replica of at least 10%. The time step was 2 fs for all simulations in solvent and 1 fs for the simulations with the solid lignin particle mimicking the powder conditions. All bonds were constrained using P-LINCS.^51^ Lennard-Jones and Coulomb cutoffs, dispersion correction, and Particle-Mesh Ewald methods were used as in the multilignin simulations.^52,53^

## Results and Discussion

### Lignin Solubility Experiments

Across the different solvents, water, ethanol, THF, acetone, and DMSO, all samples exhibited solubility ranging from partial to nearly complete at a concentration of 2 wt %. The soluble fraction of industrial lignins (softwood kraft and hardwood IEL) and model compounds (β-O4′ trimer and 5-5′ tetramer) are illustrated in Figure 3a and b, respectively. For all samples, the highest soluble fractions were achieved in DMSO, whereas water produced the lowest.

**Figure 3:**
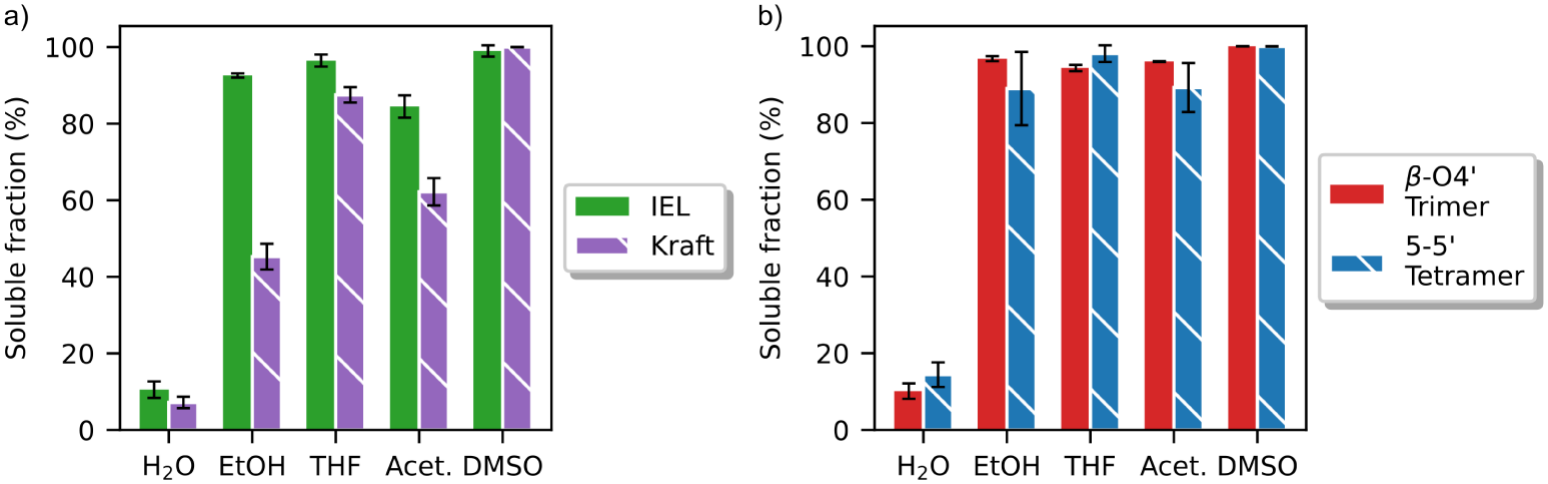
Experimentally measured soluble fractions of a) softwood kraft lignin (Kraft) and hardwood poplar lignin (IEL), as well as b) lignin model compounds trimer and tetramer.

When the industrial solvents are compared, the trend of IEL lignin dissolution is slightly different from kraft lignin (Figure 3a). Both samples show low solubility in water and high solubility in DMSO; however, the soluble fraction in the other organic solvents is higher for the IEL sample. The soluble fraction measured for the kraft lignin and the respective ranking of solvents corresponds well with previous measurements.^23^ The difference between kraft and IEL lignin, notably the higher soluble fraction compared to kraft lignin, could be due to the lower molecular weight, the ratio of G versus S units, as well as the higher prevalence of β-O4′, β-β′, and β-5′ side chain structures of IEL (Table S1). While S-unit enrichment increases methoxylation relative to G-units, numerous studies indicate that higher S content generally enhances solubility in polar organic solvents, such as ethanol, THF, and DMSO, and influences their fractionation behavior.^9,56^ In contrast, the solubility in neutral water remains low for both lignin types and is governed primarily by ionic functionality rather than the G/S ratio.^28,57,58^

The soluble fractions recorded for the trimeric and tetrameric model compounds mirror the observed trends, with DMSO and water on opposite ends of the solvent ranking (Figure 3b). Both show higher soluble fractions in ethanol, THF, and acetone than the industrial samples, consistent with their lower molecular weight enhancing solubility in these solvents.

### Multilignin Simulations

Molecular Dynamics (MD) simulations provide atomistic insight into how lignin interacts with itself and various solvents. The results presented in this section are based on simulations of several lignin molecules; each simulation contained one lignin type with differences in molecular weight and linkage composition in the solvents (Figure 1).

In water simulations, lignin consistently separated from the solvent during the equilibration phase, regardless of the molecular weight and linkage composition, as shown in snapshots in Figure 4. Although self-aggregation of lignin occurs in all simulations with water, the smaller molecules (monomers and dimers) show some lignin molecules separating from the cluster, suggesting a higher solubility for molecules with lower molecular weight (Figures S11–S13). The formation of clusters in water contrasts with the results of simulations in organic solvents, where lignin stayed dispersed in the solvent (Figure 4). Analysis of the distance between two lignin molecules via the radial distribution function (Figure S14) confirms the formation of clusters in water. Additionally, the average distance between the lignin molecules in DMSO is larger compared to those in the other organic solvents, which cannot be explained by the volume of the box.

**Figure 4:**
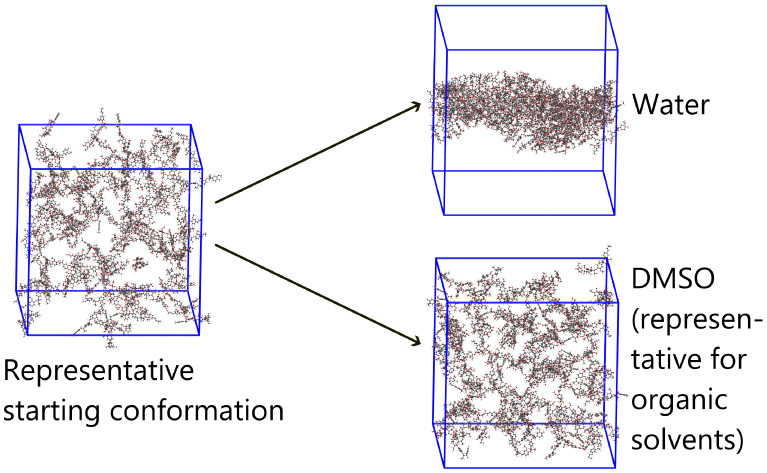
Representative simulation snapshots before energy minimization and of production runs of β-O4′ tetramer in H_2_O and DMSO. Snapshots of all simulations can be found in Figures S11 to S13.

#### Influence of Molecular Weight on Interactions in Water

In this section, the effect of lignin molecular weight on π–π stacking and hydrogen bonding interactions is investigated. The tested lignin is a sequence of repeating β-O4′ linked G units, hence results are presented as a function of the number of G units, or rings, directly proportional to molecular weight.

A special molecular association is π–π stacking between two aromatic rings, which in lignin can occur between rings within the same molecule (intramolecular) or between different molecules (intermolecular). Two geometries were considered: sandwich (parallel alignment, including laterally displaced rings) and T-shaped (orthogonal orientation between the ring planes). The number of stackings is defined as the average per ring, hence the maximum possible value is two—one on each side of the aromatic plane.

For the series of β-O4′ linked lignin molecules, the results are presented in Figure 5. Intramolecular sandwich stacking is the dominant structural feature in all systems (except the monomer) and increases with the number of rings, plateauing between the tetramer and octamer. In contrast, intermolecular sandwich and T-shaped stackings decrease with increasing molecular weight, indicating that larger lignin molecules tend to interact more within themselves rather than with other molecules. The convergence of the number of all types of stackings is observed between the numbers recorded for both the tetramers and octamers, indicating a trend for larger molecules.

**Figure 5:**
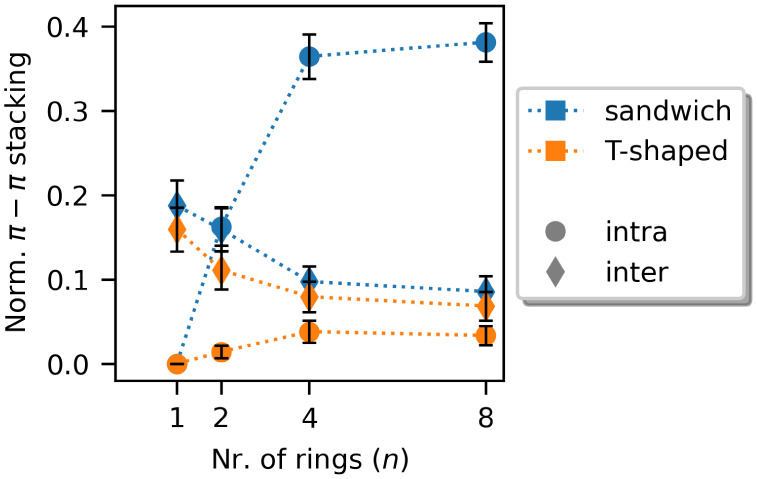
Number of intra- and intermolecular sandwich and T-shaped stacking occurrences in water, normalized with the number of rings (*n*) in the simulation. Values for thedimer, tetramer and octamer were taken from ref 30.

Hydrogen bonds are formed between a hydrogen bond donor and an acceptor. Lignin molecules included in the study have 2 donors and between 3 and 3.875 acceptor groups per phenylpropyl unit (*n*), increasing with the size of the molecule (Tables S2–S3). Lignin–lignin (LIG–LIG) hydrogen bonds are considered without distinguishing whether they occur intra- or intermolecularly.

The total number of hydrogen bonds per ring is consistent across the tested molecules, ranging from 3.3 to 3.4. The numbers of lignin–solvent and lignin–lignin hydrogen bonds converge with the larger number of lignin molecules (Figure 6). In the monomer, a lower number of hydrogen bonds between lignin molecules and a higher number between lignin and solvent are observed. This exception, compared to larger lignin molecules, is likely due to the small size of the molecule. Simulation with monomers showed more molecules breaking off from the main cluster (Figure S11). and This explains the increased interaction with the solvent rather than with other lignin molecules. Another difference between the monomer and other tested lignin molecules is the absence of a linkage to a second phenylpropyl unit. As a result, intramolecular hydrogen bonding is impossible, and all lignin–lignin hydrogen bonds in monomers are necessarily intermolecular.

**Figure 6:**
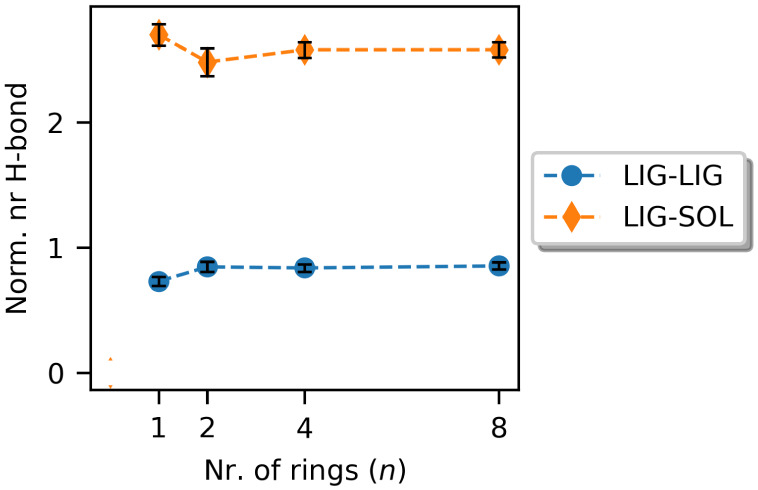
Number of hydrogen bonds normalized with the number of rings (*n*) in the lignin molecule. LIG–LIG refers to inter- and intramolecular hydrogen bonds of lignin. LIG–SOL refers to hydrogen bonds between lignin and solvent.

Analysis of π–π stacking and hydrogen bonding confirms that lignin clusters in water, regardless of molecular weight, and forms these interactions between lignins in the cluster and with water. Both π–π stacking and hydrogen bonds plateau at the tetramer size, making it a suitable and convenient model for studying these interactions and are therefore used in the comparison between solvents.

#### Influence of Solvent Condition on Interactions

In this section, simulations of lignin tetramers in different solvents are compared, focusing on π–π stacking and hydrogen bonding.

When solvents were compared, the highest total number of lignin π–π stackings was observed in water, followed by ethanol, acetone, THF, and DMSO, with the lignin in organic solvents showing significantly fewer stackings (Figure 7). In all solvents, intramolecular sandwich stackings were the most commonly observed interaction. In water, the intermolecular interactions contributed to the large total number, whereas in organic solvents, such interactions were rare. T-shaped intramolecular stackings occurred at similar levels across all solvents. Comparing the β-O4′ and 5-5′ lignin tetramers, a significant difference could only be seen in the number of intramolecular sandwich stacked occurrences in water. Here, a higher number was seen in the β-O4′ tetramer due to its higher flexibility.

**Figure 7:**
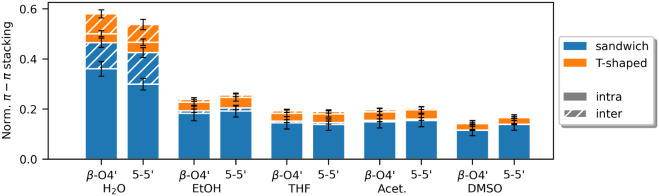
Number of intra- and intermolecular sandwich and T-shaped stacking occurrences normalized by the number of rings in β-O4′ and 5-5′ tetramers (both *n* = 4).

In the chosen solvents, water and ethanol can act both as donors and acceptors, while THF, acetone, and DMSO only have groups that can act as hydrogen bond acceptors (Tables S2–S3).

Hydrogen bonds per ring between lignin–lignin and lignin–solvent in all studied systems are presented in Figure 8. The lignin molecules were more prone to forming hydrogen bonds with the solvent than to forming intramolecular hydrogen bonds or bonds with other lignin molecules. Comparing the solvents, the number of lignin–lignin hydrogen bonds decreased in the order of water, THF, acetone, ethanol, and DMSO, similar to the ranking based on solubility.

**Figure 8:**
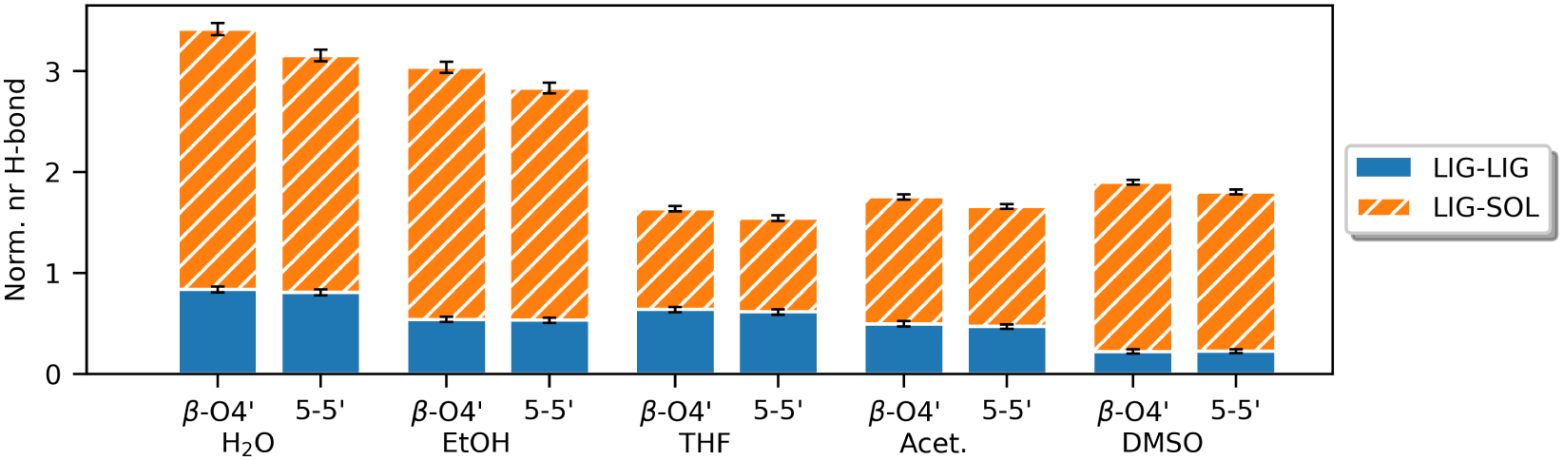
Number of hydrogen bonds normalized by the number of rings in β-O4′ and 5-5′ tetramers (both *n* = 4). LIG–LIG refers to inter- and intramolecular hydrogen bonds of lignin. LIG–SOL refers to hydrogen bonds between the lignin and solvent.

In water and ethanol, around three hydrogen bonds per ring are formed with the solvent, followed by DMSO, acetone, and THF. There was a high number of hydrogen bonds between lignin and water, though the average lignin molecule has fewer neighboring solvent molecules due to the cluster formation; see lignin–solvent RDF (Figure S14). In contrast to the lignin-lignin hydrogen bonds, there is no correlation between the number of lignin–solvent hydrogen bonds and the solubility ranking of the solvents seen in the experiments. Instead, lignin forms the highest number of hydrogen bonds with water, the poorest solvent, leading to the observation that the solubility is not directly correlated with the number of hydrogen bonds between lignin and solvent. There are several contributions to the number of lignin-solvent hydrogen bonds, such as the protic or aprotic nature of the solvent, the size of the solvent molecule, the extension of the lignin molecule, and its accessibility that likely determine the number of formed hydrogen bonds.

Comparing the two tetramers, there are more hydrogen bonds with β-O4′ tetramers than with 5-5′ tetramers in all solvents, likely due to the latter having one less hydrogen bond acceptor.

### Free Energy of Solvation

Free energy of solvation simulations were performed on single lignin molecules to quantify the cost or gain in free energy required to transfer the lignin molecule from a completely decoupled gas state to different solvents. The solvation free energies, $\Delta G_{\mathrm{solv}}^{\mathrm{solvent}}$ , are large and negative, reflecting the fact that the solvated state is always more favorable than the vapor phase (Table S4), irrespective of the solvent. As expected, water, being the worst experimentally tested solvent, gave the least negative value. For the sake of a more intuitive comparison between solvents, the transfer free energy $\Delta G_{\mathrm{solv}}^{\mathrm{aq}}$ is presented. It was calculated as the difference between $\Delta G_{\mathrm{solv}}^{\mathrm{solvent}}$ and water $\Delta G_{\mathrm{solv}}^{\mathrm{water}}$ and quantifies the change in free energy when moving from water to an organic solvent (Figure 2). A negative $\Delta G_{\mathrm{solv}}^{\mathrm{aq}}$ indicates a preference for being in the organic solvent instead of water.

Figure 9 shows the transfer free energies $\left(\Delta G_{\mathrm{solv}}^{\mathrm{aq}}\right)$ for the β-O4′ and 5-5′ tetramers in various solvents. The result enables ranking of the solvents, which are in agreement with the previous experimental and simulation results. Water was always ranked as the worst solvent and DMSO as the best solvent, while ethanol, THF, and acetone gave similar results. The difference between water and organic solvents was larger for the 5-5′ tetramer and especially pronounced in ethanol. This is partially due to a more favorable $\Delta G_{\mathrm{solv}}^{\mathrm{water}}$ for the all-β-O4′ linked tetramer (Table S4). Notably, even a minor difference in chemical structure (Figure S6) leads to a measurable difference in the free energy of solvation and transfer free energy. The more flexible β-O4′ linkage is advantageous and allows more favorable interactions between the tetramer and water, partially explaining the difference.

**Figure 9:**
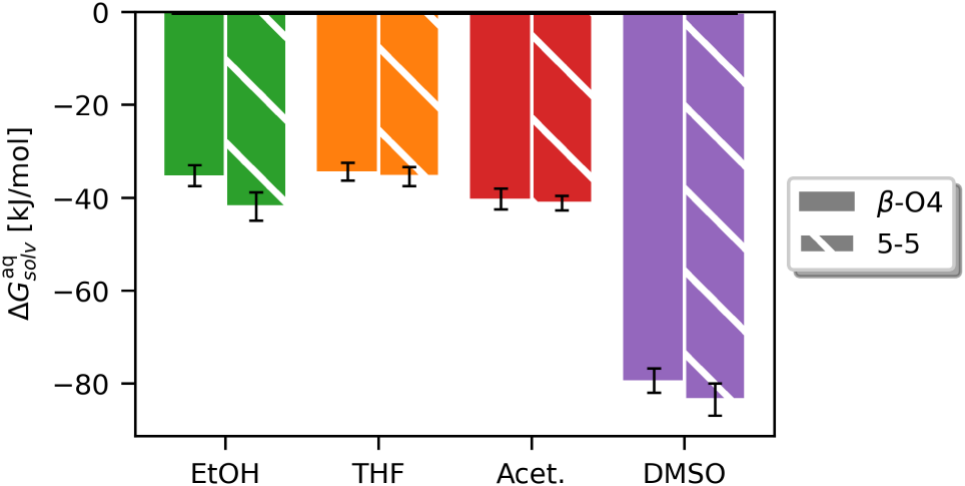
Transfer free energy of β-O4′ and 5-5′ tetramers from water to various organic solvents.

The ranking of solvents based on the results of the β-O4′ tetramer is consistent with the transfer free energies obtained with the monomers, dimers, and octamers (Figure 10). For all molecular weights, the energy was the most negative in DMSO and is nearly indistinguishable from those of the other organic solvents. If the transfer free energy is normalized with the number of rings, with increasing molecular weight, an increasingly smaller negative contribution per ring can be seen. This trend shows tapering at the largest systems. However, the curves do not level off completely, indicating that larger oligomers would be needed for the values to converge to the macromolecular limit. However, the trend of a negative contribution per ring indicates an increasing free energy difference between the organic solvents and water, but the addition of a phenylpropyl unit contributes less in a large molecule compared to a small molecule.

**Figure 10:**
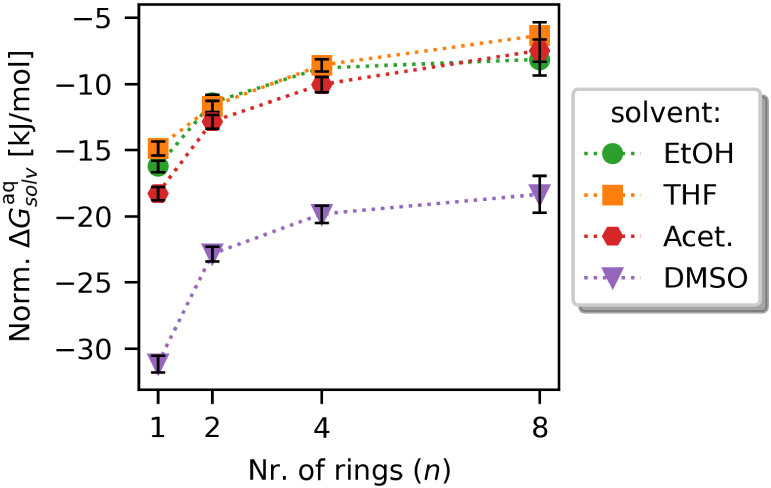
Transfer free energy for transfer of lignin from water to organic solvents. The energy is normalized per number of rings.

Free energies of solvation for monolignols and H–H dimers in organic solvents have been previously calculated by Vermaas et al., and a comparison with results from the present study is provided in Tables S5–S6.^28^ Comparing the total free energies obtained with the G monolignols and H–H β-O4′ dimers in the reference with the G–G β-O4′ dimer presented here, it is found that all $\Delta G_{\mathrm{solv}}^{\mathrm{solvent}}$ are within the error and differ by less than 5%.

#### Entropic and Enthalpic Contributions

The transfer free energy consists of an enthalpic $\left(\Delta H_{\mathrm{solv}}^{\mathrm{aq}}\right)$ and an entropic $\left(\Delta S_{\mathrm{solv}}^{\mathrm{aq}}\right)$ contribution, according to the definition of Gibbs free energy $\Delta G_{\mathrm{solv}}^{\mathrm{aq}}=\Delta H_{\mathrm{solv}}^{\mathrm{aq}}-T\Delta S_{\mathrm{solv}}^{\mathrm{aq}}$ , where *T* is the absolute temperature. The entropic contribution is only composed of the conformational entropy of lignin and solvent, respectively, and does not include any concentration-dependent part. The free energy of solvation simulations were performed with REMD at various temperatures, and the enthalpic and entropic contributions were obtained by linear fitting of $\Delta G_{\mathrm{solv}}^{\mathrm{aq}}$ vs *T* using least-squares regression, as shown in Figure S15.

For the β-O4′ tetramer (Figure 11), the difference in enthalpic contributions $\left(\Delta H_{\mathrm{solv}}^{\mathrm{aq}}\right)$ is negative, favoring organic solvents over water, whereas the difference in entropic contributions $\left(-T\Delta S_{\mathrm{solv}}^{\mathrm{aq}}\right)$ is positive and counteracts solvation in comparison to water as the solvent.

**Figure 11:**
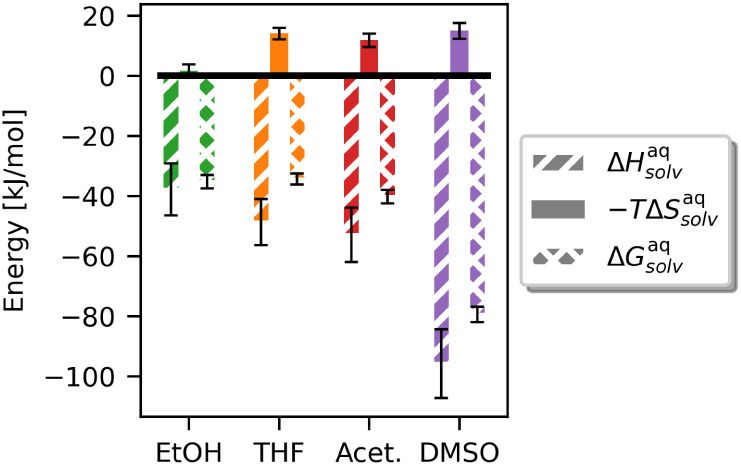
Transfer free energies for β-O4′ tetramer in organic solvents relative to water and their respective enthalpic and entropic contributions.

As a conclusion, the more favorable solvation free energy in organic solvents is primarily driven by enthalpic gains that offset the entropic penalty. This is most notable for DMSO, which has the most favorable enthalpy contribution. In ethanol, the relative entropic penalty is almost negligible, but the moderate enthalpic gain compared with water hampers the solvent efficiency.

When investigating dependence of $\Delta G_{\mathrm{solv}}^{\mathrm{aq}}$ on the number of rings, a trend of decreasing enthalpic contribution favoring solution per ring with increasing molecular weight was seen (Figure 12a). This trend is most pronounced in DMSO, for which the enthalpy quickly converges to a constant contribution per ring in the tetramer.

**Figure 12:**
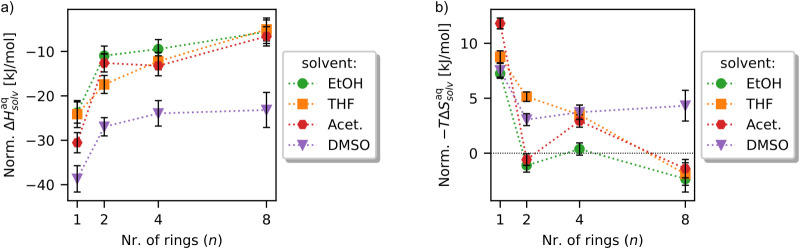
a) Enthalpic and b) entropic contributions to the transfer free energy normalized with the number of rings in various solvents.

In contrast, the entropic contribution is less consistent, with some positive and some negative values relative to water (Figure 12b). Ethanol has the lowest contribution among all tested molecules. The monomer shows an entropic penalty compared to water in all solvents, whereas the octamer has an entropic gain per ring compared to water in most solvents except DMSO. A general decrease in normalized entropy with increasing molecular weight can be seen, though this trend is not as pronounced as it was in the enthalpic contribution. The entropic term only contains conformational terms of solute and solvent and is not concentration-dependent. It is likely largely dominated by solvent entropy and related to the order the solvent has to maintain at the solute surface. From the solvent-accessible surface area (SASA), it can be seen that the surface area per ring decreases with increasing molecular weight, indicating that the generally increasing entropy is due to a decreasing normalized SASA (Figure S16). The difference between the octamers in organic solvents could be due to an additional contribution of the solute entropy, as the octamer in DMSO shows less flexibility compared to other organic solvents.^28^

Comparing the results of the free energy of solvation calculations with the simpler multilignin simulations and the experiments, the same trends in solubility are seen there. In all three approaches, water is the worst and DMSO is the best solvent. The difference in $\Delta G_{\mathrm{solv}}^{\mathrm{aq}}$ grows with increasing molecular weight, showing indications of convergence in contribution per ring. The enthalpic component of the transfer free energy is larger than the entropic contribution, assuming a near-infinite dilution.

### Estimating the Soluble Fraction Based on Calculated Free Energies

Up to this point, experimental and simulated results were compared via rankings. As a next step, a strategy was developed to obtain a shared metric, the soluble fraction in a solution with a defined lignin content. Absolute solubility can be calculated from the free energy of solvation in combination with the free energy of sublimation.^59,60^ This approach assumes infinite dilution of the saturated solution, i.e. obeying Henry’s law. This assumption is motivated by the very low concentrations in the experimental work. From the solubility, the soluble fraction of 2 wt % is calculated, which is the metric presented in the experimental section of this paper.

Additional calculations of the free energy of sublimation are added, creating the thermodynamic pathway from the solid particle mimicking the powder to the solvated state (Figure S17). In brief, additional free energy calculations were performed to decouple all interactions in a powder state (Figure S18), providing the energy needed to sublimate a single lignin molecule from a pure lignin particle, Δ*G* ^particle^ (Table S4). Due to computational limitations, only equilibrated particle states for monomers, dimers, and tetramers were obtained.

To determine the soluble fraction as measured experimentally, the free energy (Δ*G* ^total^) has to include the concentration-dependent entropy term (eq 1). *V*_1_ and *V*_2_ are the volumes per molecule in the “powder reference state” and solution, respectively. The volumes are equivalent to the inverse of the molarity. At maximum lignin concentration, aka the solubility, thermodynamic equilibrium (Δ*G* ^total^ = 0) is reached. Equation 1 can be used to calculate the volume per lignin molecule in the solution, which gives the maximum concentration in weight percent that can be achieved. The soluble fraction is the ratio *f*(2 wt %) between the maximum and 2 wt %, eq 2:

1 $$\Delta G^{\mathrm{total}}=\Delta G_{\mathrm{solv}}^{\mathrm{solvent}}-\Delta G^{\mathrm{particle}}-k_{B}T\ln\left(\frac{V_{2}}{V_{1}}\right)$$

2 $$f\left(2\;\mathrm{wt}\;\%\right)=\frac{\max\mathrm{wt}\;\%}{2\;\mathrm{wt}\;\%}\times 100\%=\frac{Mw}{\rho V_{2}N_{A}}\frac{1}{2\;\mathrm{wt}\;\%}\times 100\%$$

Soluble fractions based on the calculation of $G_{\mathrm{solv}}^{\mathrm{particle}}$ for all lignin molecules and solvent combinations in a 2 wt % solution are presented in Table 1. As expected, the solubility decreases with increasing molecular weight, and the monomer was calculated to be 100% soluble at a concentration of 2 wt % in all solvents, the dimer in all organic solvents, and the tetramer only in DMSO. The calculated soluble fractions of the 5-5′ tetramer are lower in all solvents, except DMSO, than that was measured experimentally. However, due to the logarithmic nature of eq 1, small variations in calculated corrected free energies give rise to large jumps in soluble fraction (Figure S19).

**Table 1:** Soluble Fractions Calculated Based on Free Energy of Solvation

	Soluble fraction in 2 wt % solution [%]			
	Monomer	Dimer	β-O4′ Tetramer	5-5′ Tetramer
Water	100	40	3 × 10^–8^	5 × 10^–9^
EtOH	100	100	0.06	0.13
THF	100	100	0.04	0.01
Acet.	100	100	0.48	0.09
DMSO	100	100	100	100

A key limitation of the approach is the difficulty in defining a representative powder state and the slow dynamics seen in lignin systems without a solvent. The system with the solid particle was chosen as the best compromise between choosing a relevant system and maintaining the feasibility of the computations in practice. However, any error introduced by these approximations will shift the results of $G_{\mathrm{solv}}^{\mathrm{particle}}$ across all solvents. Additionally, there are also minor differences in the chemical structure of the 5-5′ lignin model between the simulation and the experiment, and due to computational limitations, the molecular weight series was limited to tetramers as the largest lignin molecules.

This approach demonstrates both the potential and the limitations of solvation free energy calculations. It was possible to calculate the same metric as experiments measure (soluble fraction), but the method is highly dependent on a well-defined solid state. This remains to be developed by both modeling and experimental efforts.

### Comparison with Other Solubility Predictions

#### COSMO-RS Calculations of Activity Coefficients

The logarithmic activity coefficients at infinite dilution ln­(γ) were calculated for lignin pentamers with varying H/G/S unit and linkage composition using COSMO-RS based on MD and DFT conformations. ln­(γ) of an all G and β-O4′ linked pentamer and an average over all 28 tested pentamers presented by Sumer et al. are listed in Table 2. The ranking of the solvents is based on a large negative ln­(γ) value, indicating likely dissolution of the solute in the solvent, whereas with a positive ln­(γ) negligible dissolution is expected. This ranking shows water as the worst solvent and DMSO as the best solvent. THF and acetone give very similar results, with flipped ranking depending on results for the all G β-O4′ pentamer and the averaged value. These solvents have a noticeably better expected solubility than ethanol. The ranking of water and DMSO agrees with the ranking presented based on the free energy of solvation calculations, whereas ethanol shows a larger distinction from acetone and THF for ln­(γ) than was seen in the free energy of solvation. Whether this difference is due to the method or the lignin composition and number of phenylpropyl units cannot be determined, and the experimental results for the lignin model compounds show no significant difference between ethanol, acetone, and THF.

**Table 2:** ln­(γ) as Presented by Sumer et al. for an All G β-O4′ Pentamer and Averaged over All Presented for 28 Pentamers as well as Their Respective Solvent Ranking^36^

	All G β-O4′ pentamer		Average	
	ln(γ)	Ranking	ln(γ)	Ranking
Water	17.3	5	13.9	5
EtOH	–0.8	4	–2.3	4
THF	–6.4	2	–8.9	2
Acet.	–6.7	3	7.7	3
DMSO	–12.1	1	–17.7	1

#### Hansen Solubility Parameters

The software HSPiP was used to estimate the solubility parameters of the lignin dimer using the Yamamoto Molecular Breaking (Y-MB) group contribution method.^61^ The calculated parameters together with tabulated solvent parameters and literature values for softwood kraft lignin^35^ are presented in Table 3. The key insight is that the difference between ideal lignin dimers and kraft lignin HSPs is minor and that water’s extremely high hydrogen bonding parameter works against lignin dissolution.

**Table 3:** Hansen Solubility Parameters for Lignin Dimer and Tetramer (Calculated), Kraft Lignin,^35^ and Solvents^61^

	Lignin			Solvents				
	Dimer	Tetramer	Kraft	Water	EtOH	THF	Acet.	DMSO
δ_d_	19.6	21.3	17.6	15.5	15.8	16.8	19.9	18.4
δ_p_	7.7	2.5	12.6	16.0	8.8	5.7	10.4	16.4
δ_h_	13.7	7.6	16.0	42.3	19.4	8.0	7.0	10.2
δ_total_	25.1	22.8	26.9	47.8	26.5	19.5	19.9	26.7

From these values, the Hansen “distance” between the solute and solvent was calculated for the lignin dimer as

$$R_{a}={\left[4{\left(\delta_{d,\mathrm{lignin}}-\delta_{d,\mathrm{solvent}}\right)}^{2}+{\left(\delta_{p,\mathrm{lignin}}-\delta_{p,\mathrm{solvent}}\right)}^{2}+{\left(\delta_{h,\mathrm{lignin}}-\delta_{h,\mathrm{solvent}}\right)}^{2}\right]}^{1/2}$$

and the relative energy difference (RED) is RED = *R*_a_/*R*_0_, where *R*_0_ is the radius of the solubility sphere. In Table 4, the RED values are presented together with a classification of the solvent as “Poor” or “Good”, since if RED < 1, the solute is soluble, and if RED > 1, it is insoluble.

**Table 4:** Relative Energy Distances (RED) between Lignin Tetramer and Solvents and Classification of Solvent Based on HSP Calculations with *R*_0_ = 10

	Water	EtOH	THF	Acet.	DMSO
RED	3.90	1.73	0.96	0.84	1.53
Classific	Poor	Poor	Good	Good	Poor

By this estimation, THF and acetone were predicted to be better solvents than DMSO, which is in contrast to both simulation and experimental results, both of which found that DMSO is not only a good solvent but also the best choice among this set of solvents.

## Conclusions

A comprehensive evaluation of lignin solubility from both experimental and simulation perspectives was conducted. The experimental results established reference solubility values and solvent rankings for two well-defined lignin model compounds, as well as for more complex industrial samples, namely kraft and IEL lignin. Free energy of solvation calculations based on molecular dynamics simulations, similar though not identical to the experimental, well-defined lignin models with varying molecular weights and linkage types, yielded solvent rankings consistent with those obtained for the experimental model compounds. Notably, kraft lignin solubility deviates from the experimental model lignin solubility, as well as from the simulation results. This is an indication that the structural differences in kraft lignin compared to native lignins are important for its solubility properties.

The simulations indicate that the higher solubility in organic solvents compared with water was mainly driven by enthalpic contributions to the transfer free energy. Analysis of hydrogen bonding between the solvent and lignin showed that although substantial hydrogen bonding between lignin and the solvent is required for dissolution, it must be in combination with other types of enthalpic interactions. The hydrogen bonding ability of a solvent alone did not correlate with its solubility ranking from the free energy of solvation calculations. Lignin–lignin interactions by π–π stacking were found in all solvents; however, in the lowest-ranked solvents, intermolecular π–π stacking was more frequent.

In summary, the close alignment between experimental results and molecular dynamics simulations regarding the lignin solubility of well-defined lignin structures underscores the effectiveness and precision of computational modeling in accurately capturing the solubility behavior of the complex molecular structures of lignin. While single parameters such as hydrogen bonding ability or molecular polarity are not sufficient, MD simulations provide a holistic view of lignin solubility and have been shown to be a useful tool toward understanding its mechanisms.

## Supplementary Material



## Notes

Additional information about experimental lignin compounds, chemical structures of lignin used in experiments and simulations, simulation snapshots, radial distribution functions, solvent-accessible surface areas, and additional information from free energy of solvation calculations (PDF)
