# Supplementary material for: Thermodynamic and Atomistic Insights into Lignin Solubility from Experimentally Validated Molecular Dynamics Simulations
Source: Biomacromolecules. 2026 Apr 21;27(6):3533–43. doi: 10.1021/acs.biomac.5c02503 (PMC13250915; doi:10.1021/acs.biomac.5c02503)
Supplement: Supplementary file 1 [file bm5c02503_si_001.pdf]

Supporting Information: Thermodynamic and atomistic insights into lignin solubility from experimentally validated molecular dynamics simulations

Klara Hackenstrass, Nissa Nurfajrin Solihat, Paula Nousiainen, Monika Österberg, Sara Florisson, Jakob Wohlert, and Malin Wohlert*

E-mail: malin.wohlert@angstrom.uu.se

Additional information about experimental lignin compounds, chemical structures for lignin used in experiments and simulation, simulation snapshots, radial distribution functions, solvent accessible surface areas and additional information from free energy of solvation calculations.

Chemical Structures of experimental lignin samples

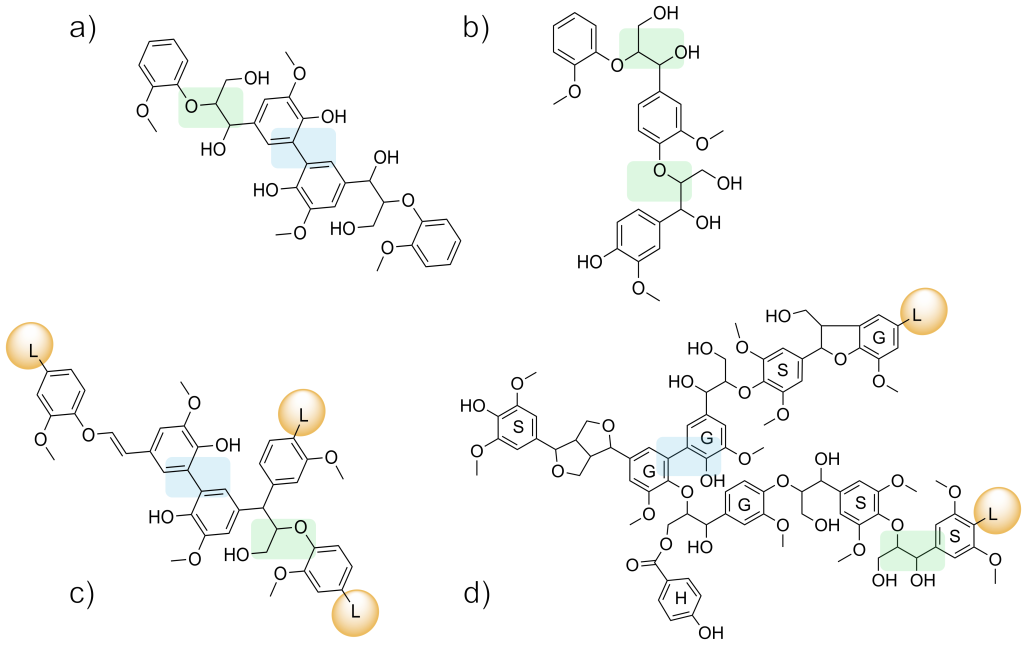

Figure S1: Lignin structures for experimental soluble fraction measurements: a) tetramer, b) trimer, illustrative structures for c) kraft and d) IEL, where L implicates lignin chains.

Structural analysis of samples by HSQC NMR

The samples used for solubilization testing were analyzed using HSQC NMR spectroscopy.

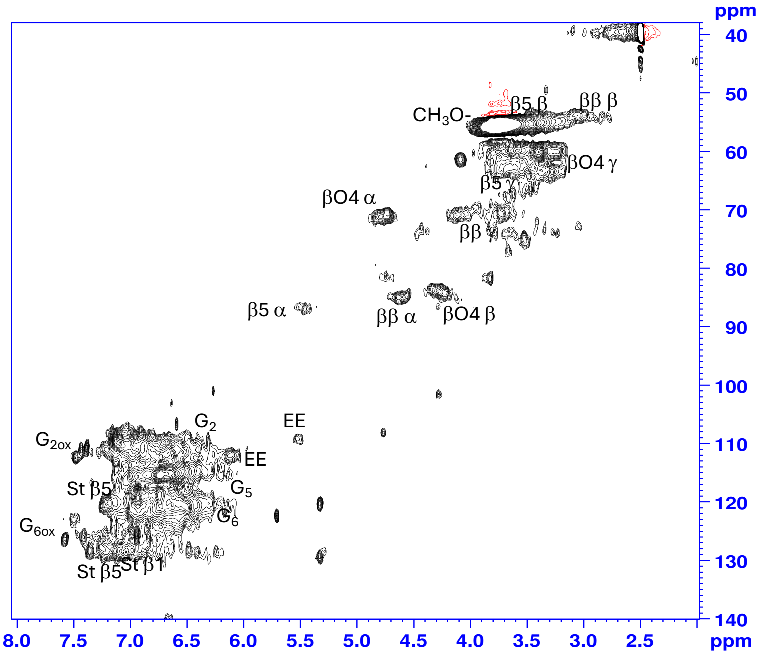

Figure S2: Softwood Kraft lignin BioPiva™ 395 (Kraft).

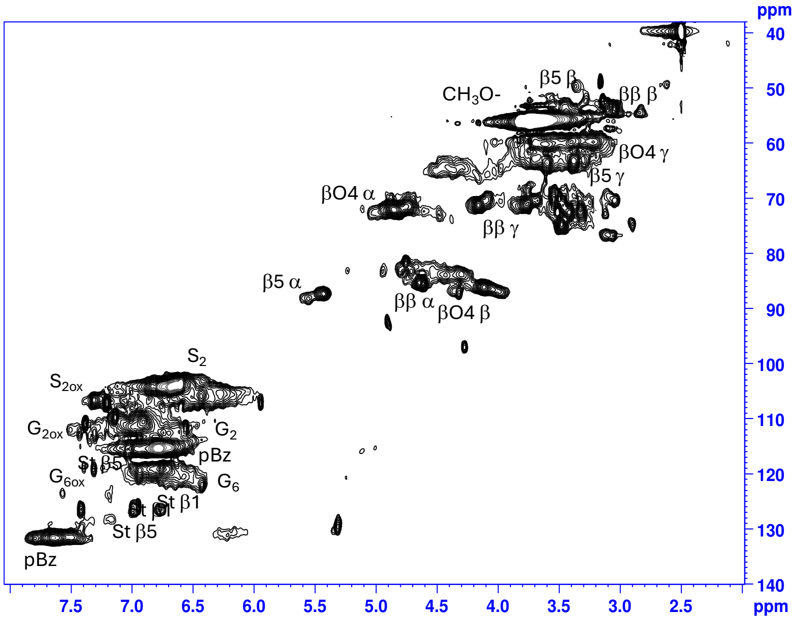

Figure S3: Ethanolic biorefinery-based hardwood poplar lignin (IEL).

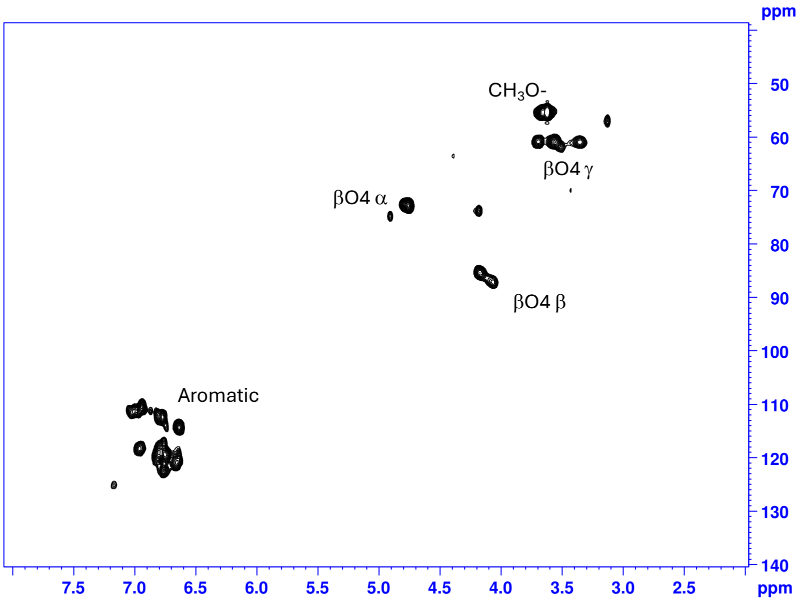

Figure S4: β-O4' trimer.

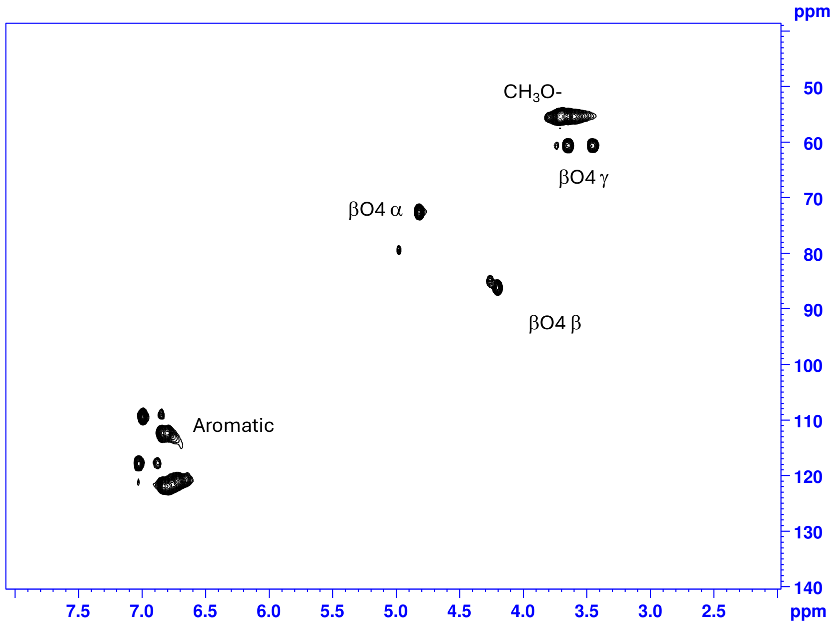

Figure S5: β-O4'-55'-β-O4' tetramer.

Chemical composition of industrial lignins

Table S1: Structural units based on their relative amounts vs. 100 aromatic units of softwood Kraft lignin (Kraft) and hardwood poplar lignin (IEL).

**Composition**	**Kraft**	**IEL**
Mn (g mol^{-1})	920	1080
Mw (g mol^{-1})	3240	2200
Đ (Mw/Mn)	3.5	2.0
**Hydroxyl groups in mmol g^{-1}**		
Aliphatic-OH	2.1	2.7
5-substituted OH/ S-OH	1.6	2.0
G-OH	1.7	1.1
H-OH	0.1	0.4
CO-OH	0.4	0.3
Total phenolic-OH	3.4	3.6
Total –OH	5.9	6.5
**Structural units described as % / aromatic group**		
Guaiacyl (G)-units	92.8	38.4
Guaiacyl oxidized (Gox)-units	4.6	6.8
Syringyl (S)-units	–	55.0
Syringyl oxidized (Sox)-units	–	5.6
p-OH-phenyl (H)-units	2.6	1.0
CH_3O-	108.1	122.3
Sidechains		
β-O4' ether	7.7	21.9
β-5' phenylcoumaran	2.8	5.1
β-β' recinol	2.5	8.2

Table S1 (continued)

**Composition**	**Kraft**	**IEL**
β-β' secoisolarisirecinol	1.3	–
Stilbene β-1'	5.0	1.6
Stilbene β-5'	6.8	3.1
Enol-ether Z	1.3	1.8
Enol-ether E	4.0	–
Terminal units		
Aldehyde-	1.3	5.3
Ferulic/ p-Coumaric acid-	1.6	1.7
p-OH Benzoic acid esters		11.6
Coniferyl alcohol-	1.8	–

Chemical structures of simulated lignin

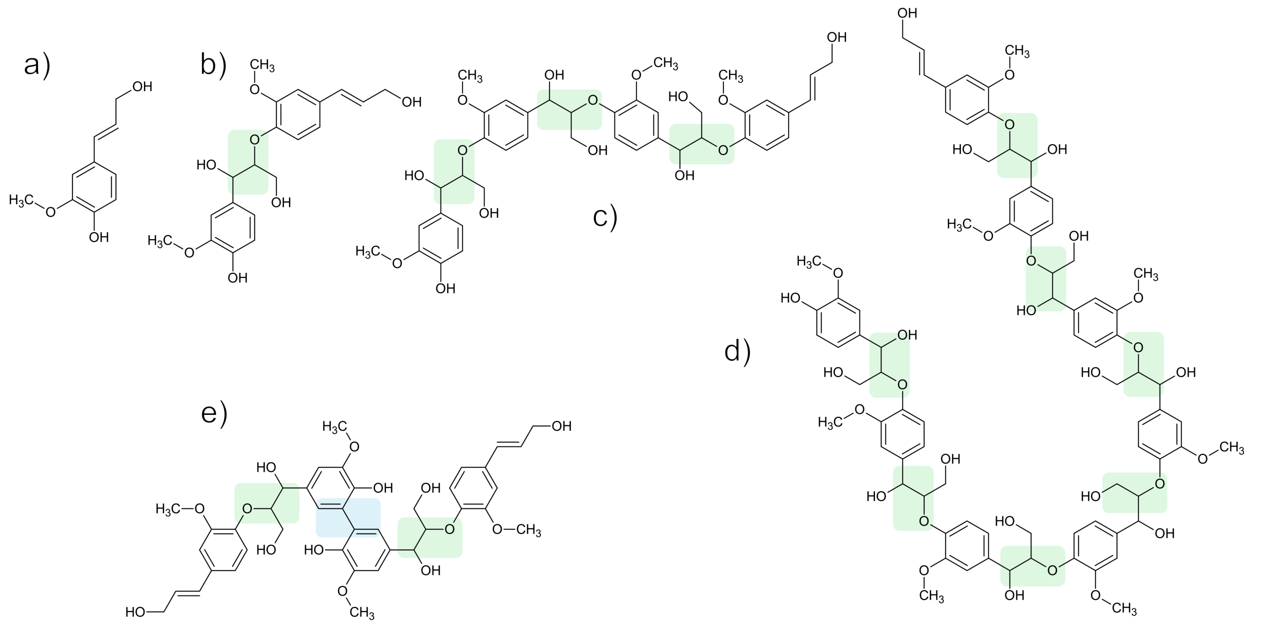

Figure S6: Lignin structures for Molecular Dynamics simulations: a) monomer, b) dimer, c) β-O4' tetramer, d) octamer and e) 5-5' tetramer.

Equilibration of simulations

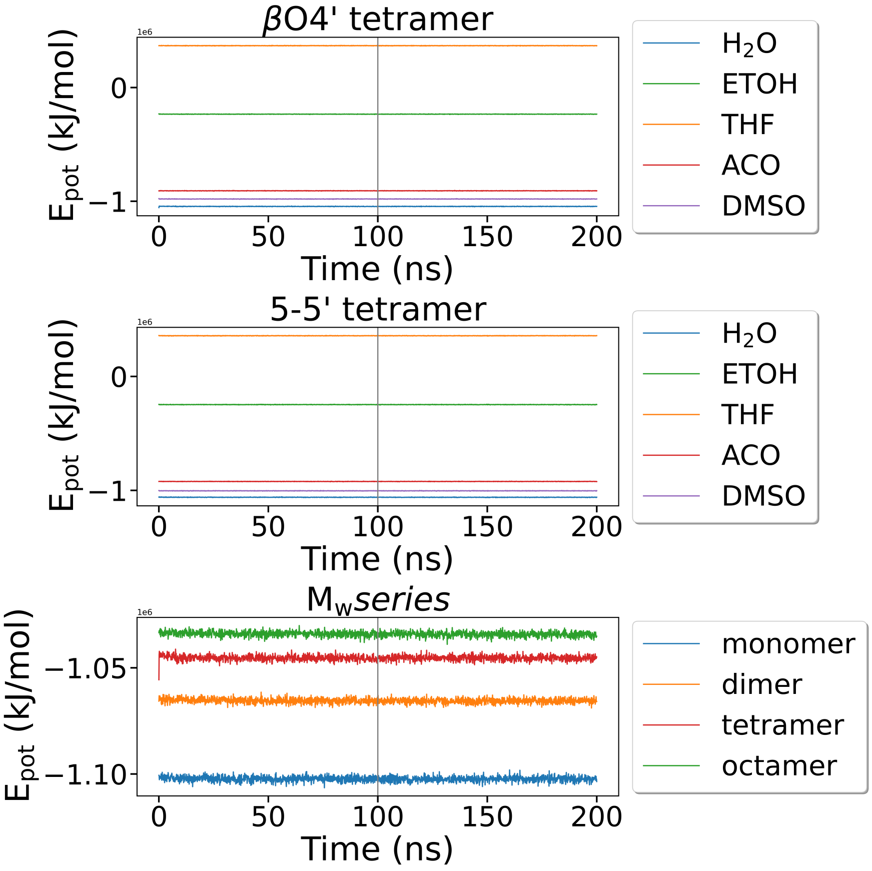

Figure S7: Potential energy versus simulation time with indicated vertical line at 100 ns. Equilibration 0 to 100 ns and production run 100 to 200 ns.

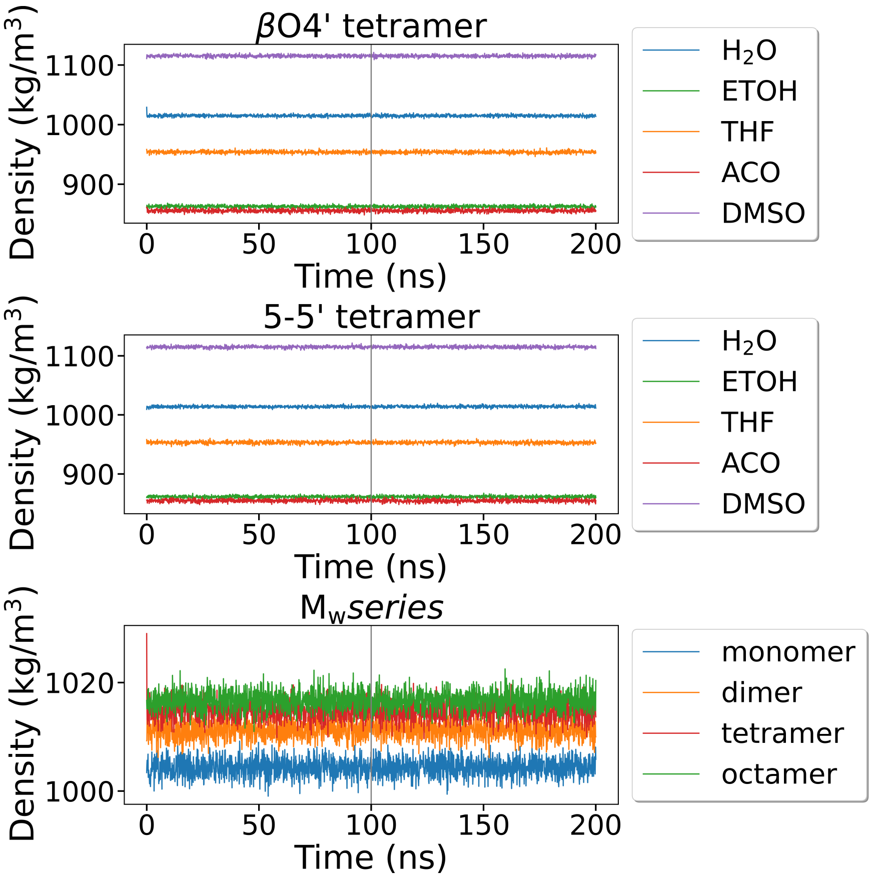

Figure S8: Combined lignin and solvent density versus simulation time with indicated vertical line at 100 ns. Equilibration 0 to 100 ns and production run 100 to 200 ns.

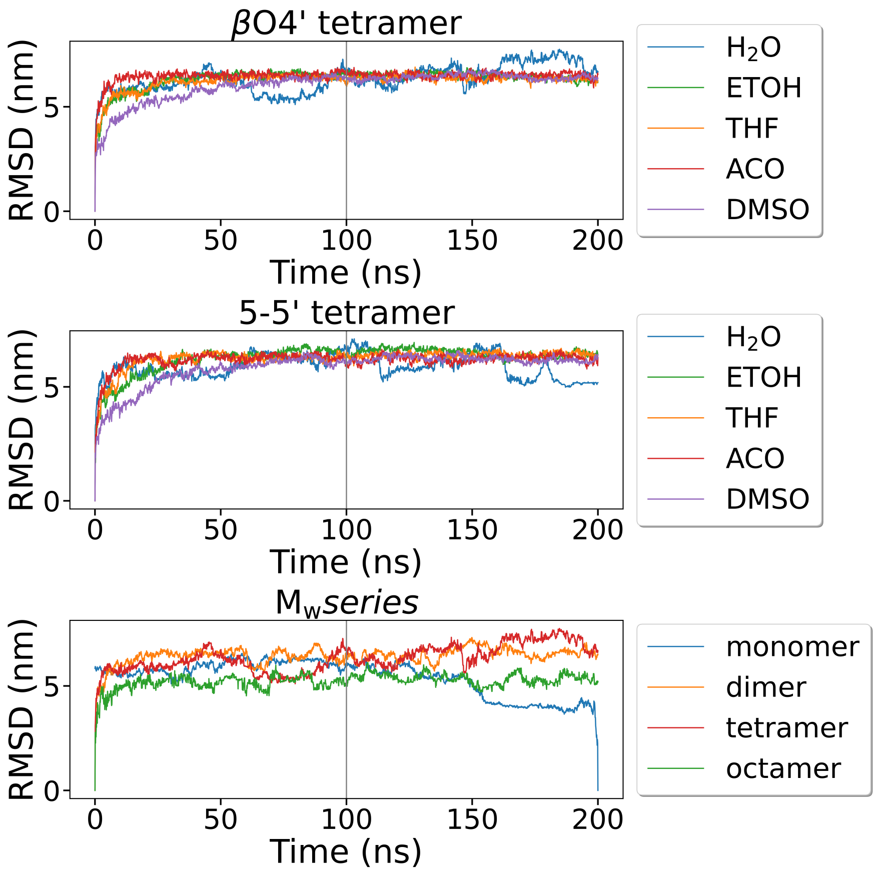

Figure S9: Root mean square displacement comparing the structure of lignin molecules compared to the initial conformation versus simulation time with indicated vertical line at 100 ns. Equilibration 0 to 100 ns and production run 100 to 200 ns.

Free energy of solvation - determining λ values

The sequence of λ_{vdW} and λ_{C} was determined based on the amount of change, increasing the resolution around areas with larger change. This approach increases the overlap between the individual states, ensuring that BAR gives correct results of the difference in $\Delta\lambda$ with low associated errors.

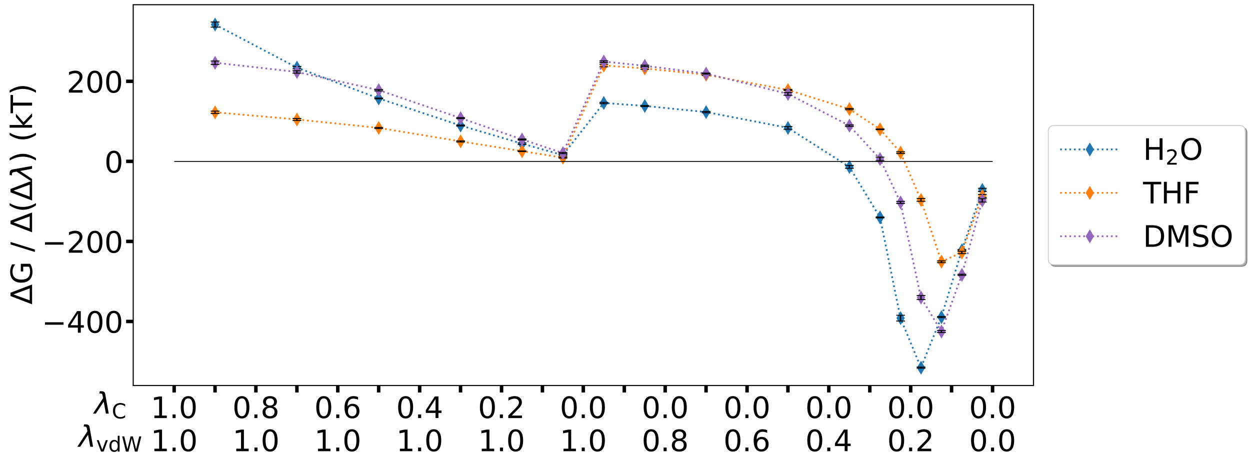

Figure S10: Change in solvation free energy normalized by the difference in $\Delta\lambda$ between neighboring values. Exemplary values and their corresponding errors shown for β-O4' tetramer in water, THF and DMSO at T=300K.

Simulation snapshots

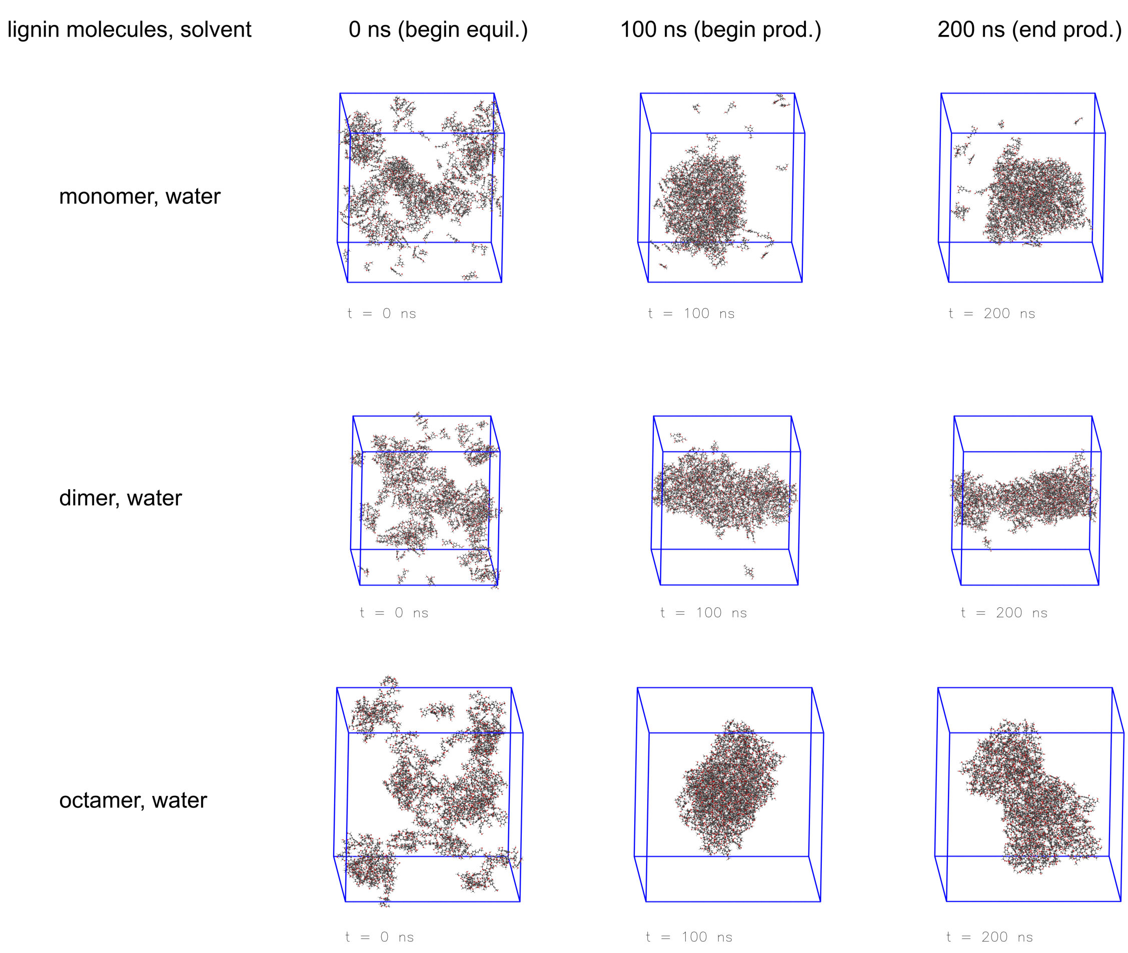

Figure S11: Molecular Weight Series: Overview over snapshots taken at the beginning of the equilibration at production run conditions as well as start and end of the production run for all systems.

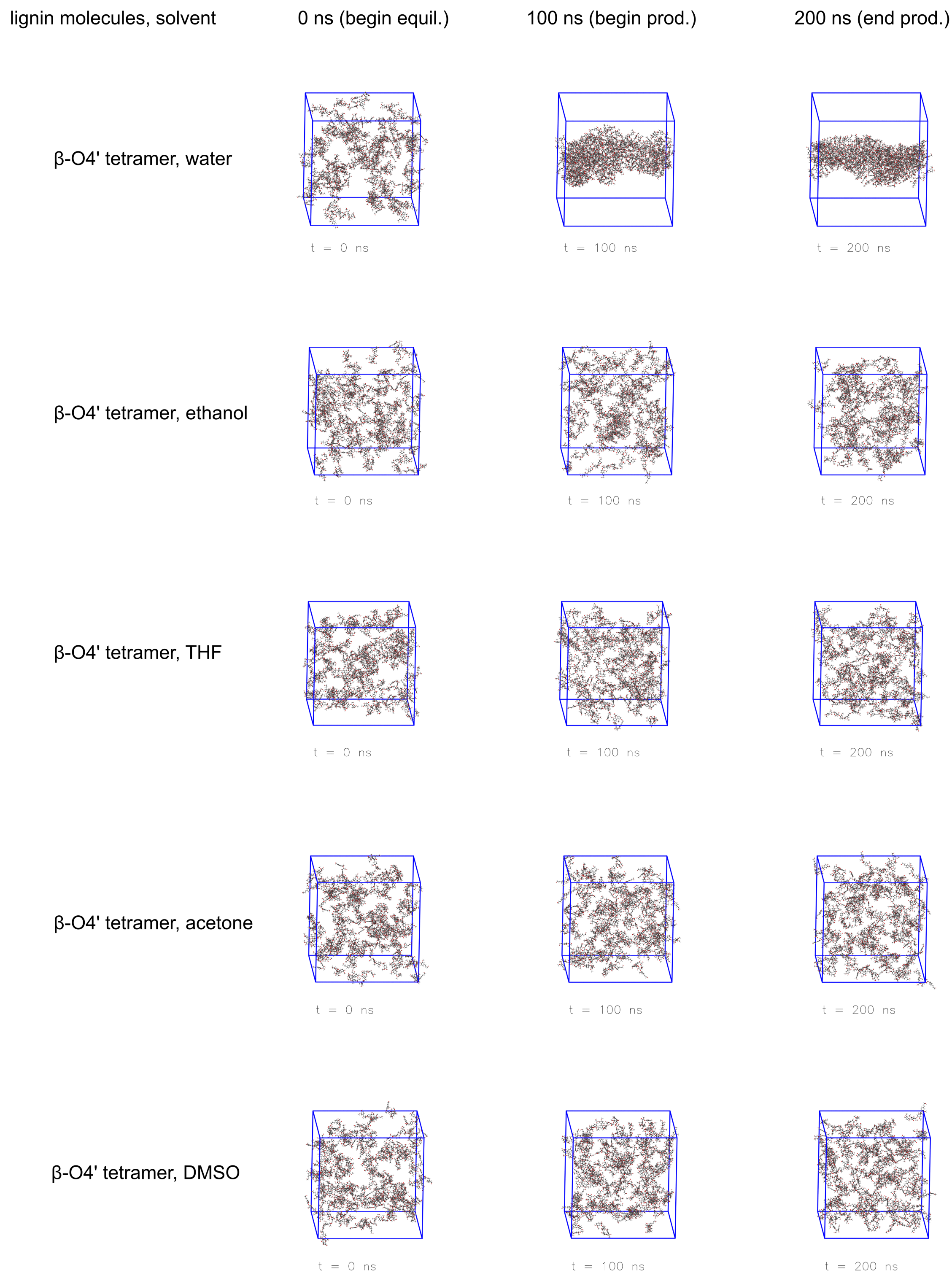

Figure S12: β-O4' Tetramer: Overview over snapshots taken at the beginning of the equilibration at production run conditions as well as start and end of the production run for all systems.

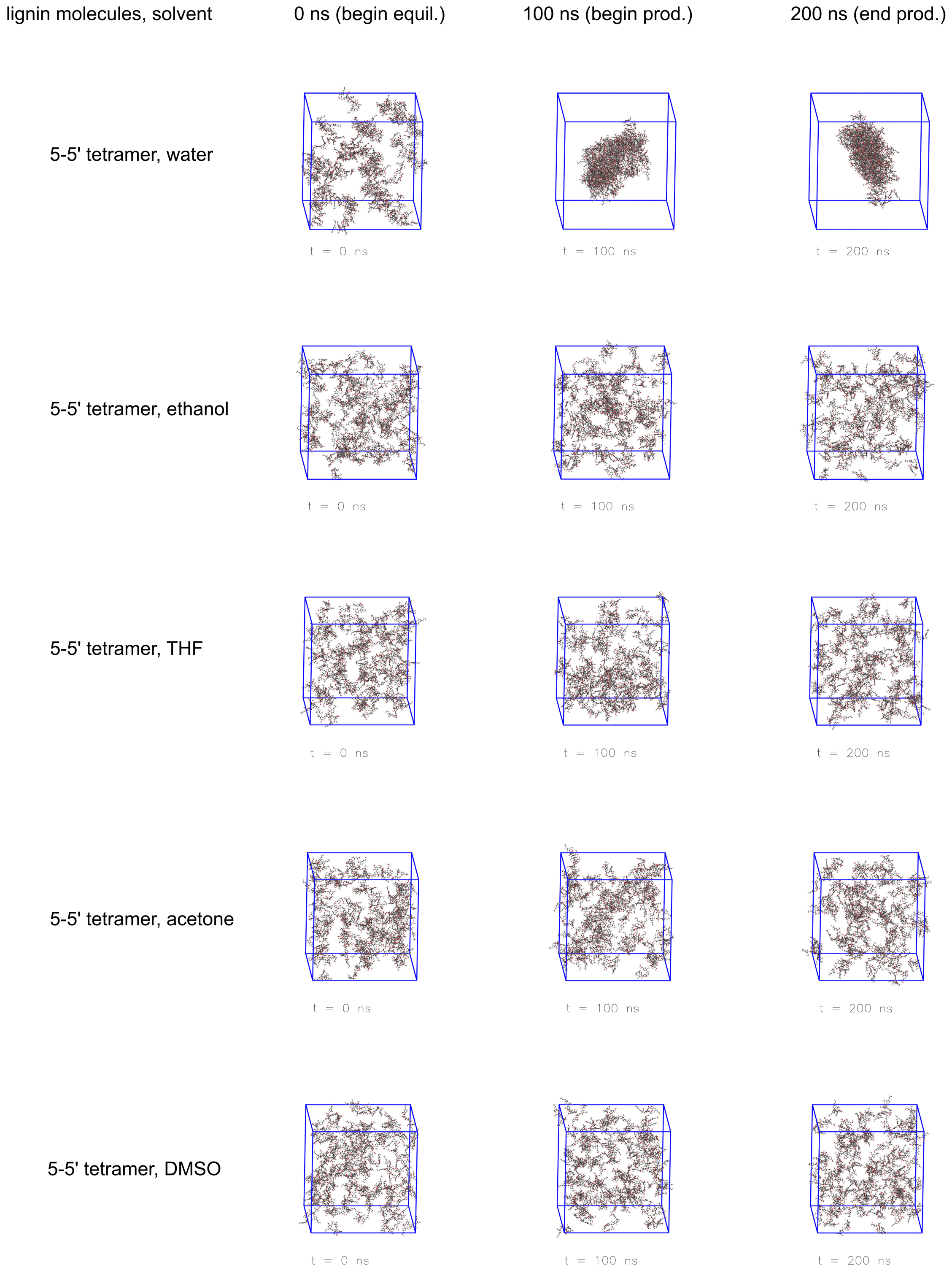

Figure S13: 5-5' Tetramer: Overview over snapshots taken at the beginning of the equilibration at production run conditions as well as start and end of the production run for all systems.

Radial distribution functions for cluster formation in solvents

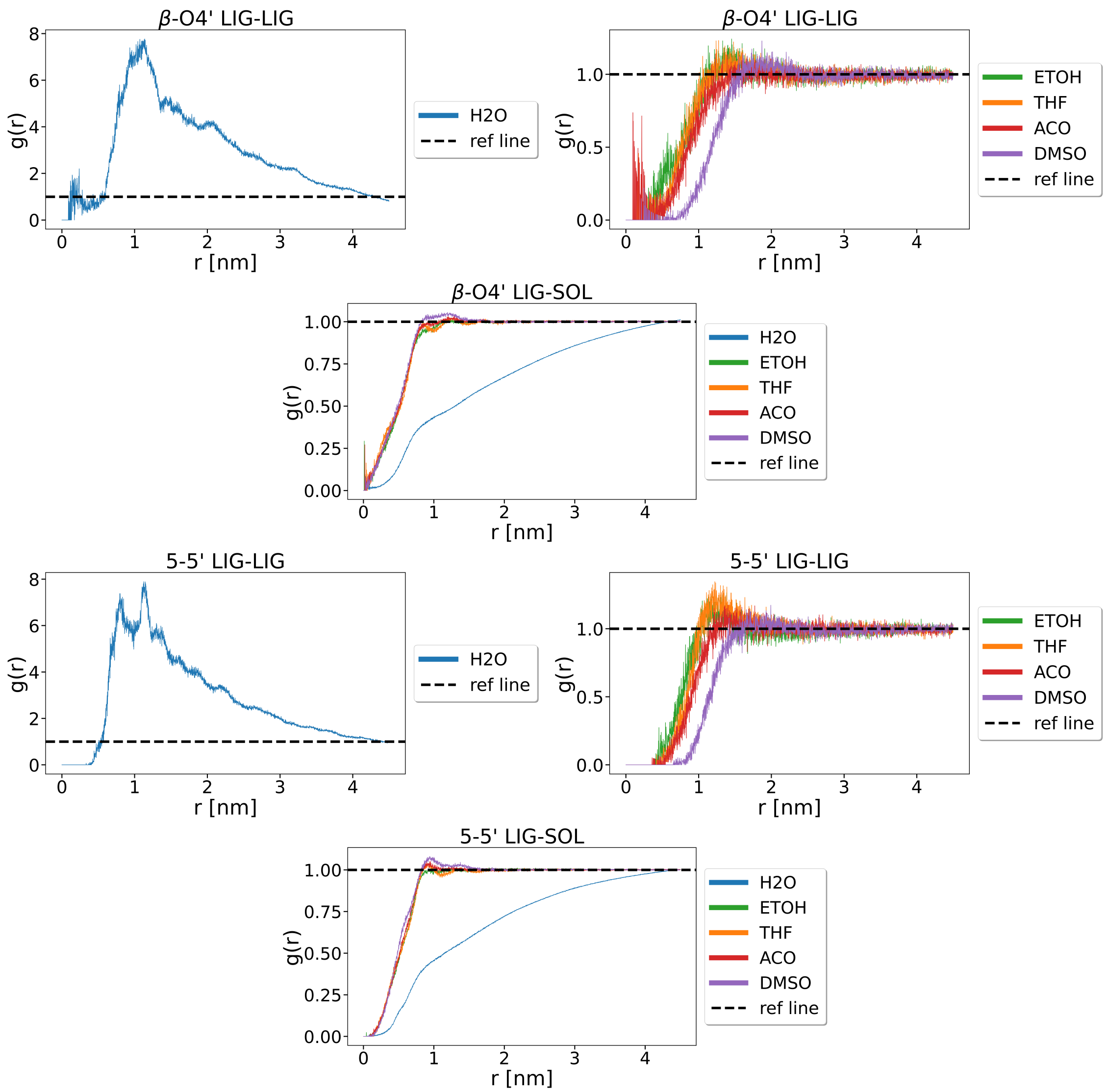

Figure S14: Radial distribution function between center of mass of lignin to other lignin (LIG-LIG) or solvent molecules (LIG-SOL). Note: The plots are normalized to the average density of lignin molecules and the distance does not cover the entire radius possible in the box. This leads to very high or low g(r) values due to the cluster formation in water.

Hydrogen bond donor and acceptor groups in lignin molecules and solvents

Table S2: Overview of number of donor (hydroxy) and acceptor (hydroxy, methoxy and ether-oxygen) groups in the simulated lignin molecules per phenylpropyl unit. Calculated by dividing the number of donor and acceptor group by the number or phenylpropyl units in the molecule.

		Donor	Acceptor	Sum
Monomer		2	3	5
Dimer		2	3	5
Tetramer	β-O4'	2	3.75	5.75
	5-5'	2	3.5	5.5
Octamer		2	3.875	5.875

Table S3: Overview of number of donor and acceptor groups in the solvents.

	Donor	Acceptor	Sum
H2O	2	1	3
EtOH	1	1	2
THF	0	1	1
Acet.	0	1	1
DMSO	0	1	1

Solvation free energies, enthalpies and entropies

Table S4: Free energy of solvation ΔG at 300K and its enthalpic ΔH and entropic $-T\Delta S$ contributions for all simulated combinations of lignin molecules and solvents.

Molecule	Solvent	ΔG [kJ/mol]	ΔH [kJ/mol]	$-T\Delta S$ [kJ/mol]
Monomer	H_2O	-47.7 (0.4)	-88.1 (1.9)	40.8 (0.4)
	EtOH	-64.0 (0.4)	-112.0 (2.4)	48.0 (0.4)
	THF	-62.6 (0.5)	-112.3 (3.1)	49.5 (0.5)
	ACO	-66.0 (0.5)	-118.7 (2.3)	52.6 (0.5)
	DMSO	-78.9 (0.6)	-126.8 (3.0)	48.3 (0.6)
	powder	-26.8 (3.0)	-59.3 (17.3)	33.6 (3.0)
Dimer	H_2O	-89.3 (0.8)	77.4 (0.8)	-165.7 (3.9)
	ETOH	-112.2 (1.4)	75.2 (1.4)	-187.6 (5.4)
	THF	-112.7 (1.0)	87.7 (1.0)	-200.6 (5.1)
	ACO	-115.1 (1.2)	76.2 (1.2)	-190.9 (5.1)
	DMSO	-135.1 (1.2)	83.5 (1.2)	-219.6 (5.2)
	powder	-96.2 (2.1)	101.3 (2.1)	-196.6 (11.7)
β-O4' Tetramer	H_2O	-154.6 (1.9)	105 (1.9)	-258.8 (7.0)
	ETOH	-189.8 (1.6)	106.5 (1.6)	-296.6 (8.0)
	THF	-189 (1.2)	119 (1.2)	-307.5 (7.1)
	ACO	-194.8 (1.6)	116.7 (1.6)	-311.7 (8.5)
	DMSO	-234 (1.9)	119.9 (1.9)	-354.5 (10.9)
	powder	-214.8 (1.5)	122.7 (1.5)	-338.2 (8.8)
5-5' Tetramer	H_2O	-147 (1.7)	86.6 (1.7)	-231.7 (5.6)
	ETOH	-189 (2.9)	114.3 (2.9)	-303.6 (7.5)
	THF	-182.4 (1.9)	102.5 (1.9)	-286.1 (7.8)
	ACO	-188.2 (1.4)	107.7 (1.4)	-297.1 (6.3)

Molecule	Solvent	ΔG [kJ/mol]	ΔH [kJ/mol]	$-T\Delta S$ [kJ/mol]
	DMSO	-230.5 (3.3)	117 (3.3)	-346.1 (12.3)
	powder	-210.8 (5.0)	114 (5.0)	-323.1 (22.6)
Octamer	H_2O	-236.5 (5.6)	224.8 (5.6)	-459.9 (16.0)
	ETOH	-301.8 (7.2)	206.1 (7.2)	-504.3 (20.6)
	THF	-287.3 (5.8)	209.7 (5.8)	-500.2 (19.9)
	ACO	-296.4 (4.4)	213.6 (4.4)	-512.6 (16.8)
	DMSO	-383.4 (8.8)	259.4 (8.8)	-645.3 (31.0)

Comparison of free energy of solvation with literature

Table S5: Difference in free energy of solvation for the same monomer presented in the current study and by Vermaas *et al.*[1] converted to kJ/mol.

solvent	presented here [kJ/mol]	literature[1] [kJ/mol]	difference [%]
H_2O	-47.7 (0.4)	-50 (8)	3.8
EtOH	-64.0 (0.4)	-62 (4)	2.4
THF	-62.6 (0.5)	-60 (4)	4.9
Acet.	-66.0 (0.5)	-65 (4)	1.7
DMSO	-78.9 (0.6)	-76 (4)	3.5

Table S6: Difference in free energy of solvation for dimers presented in the current study and by Vermaas *et al.*[1] converted to kJ/mol. Note the difference in the structure, the currect study uses a β-O4' linked G-G dimer wheras Vermaas *et al.* used the β-O4' linked H-H dimer.

solvent	presented here [kJ/mol]	literature[1] [kJ/mol]	difference [%]
H_2O	-89.3 (0.8)	-85 (8)	4.5
EtOH	-112.2 (1.4)	-109 (8)	2.5
THF	-112.7 (1.0)	-108 (4)	4.4
Acet.	-115.1 (1.2)	-115 (4)	0.2
DMSO	-135.1 (1.2)	-131 (8)	2.7

Linear Regression for REMD

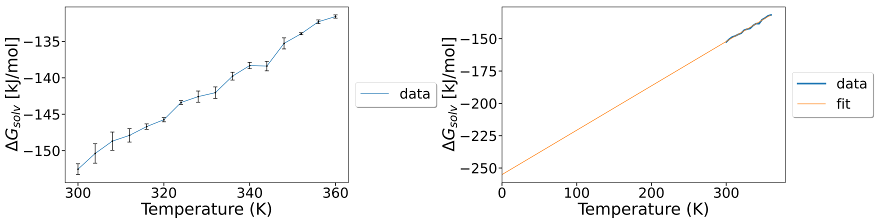

Figure S15: Example of ΔG vs T of β-O4' tetramer in water. Left: ΔG and error values in simulated temperature range. Right: ΔG and fit, y-intersect corresponds to ΔH and the slope -ΔS.

Solvent accessible surface area (SASA)

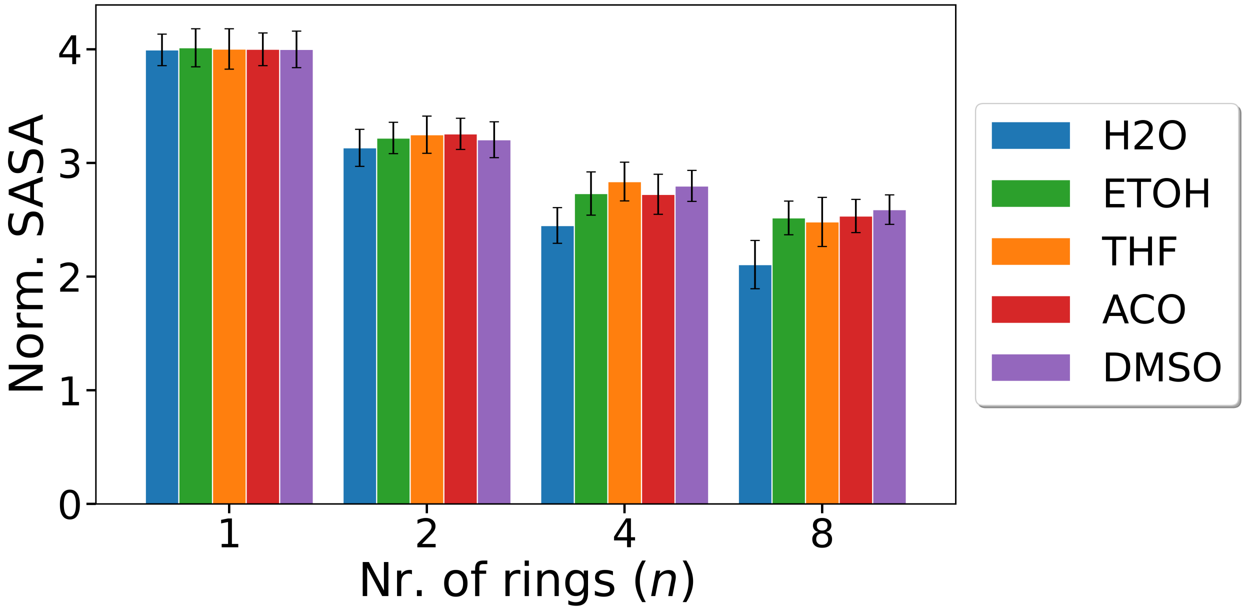

Figure S16: Solvent accessible surface area normalized with the number of rings (n) calculated using *gmx sasa* using the standard probe sphere radius of 1.4 Å.

Calculation of soluble fraction from free energy of solvation calculations

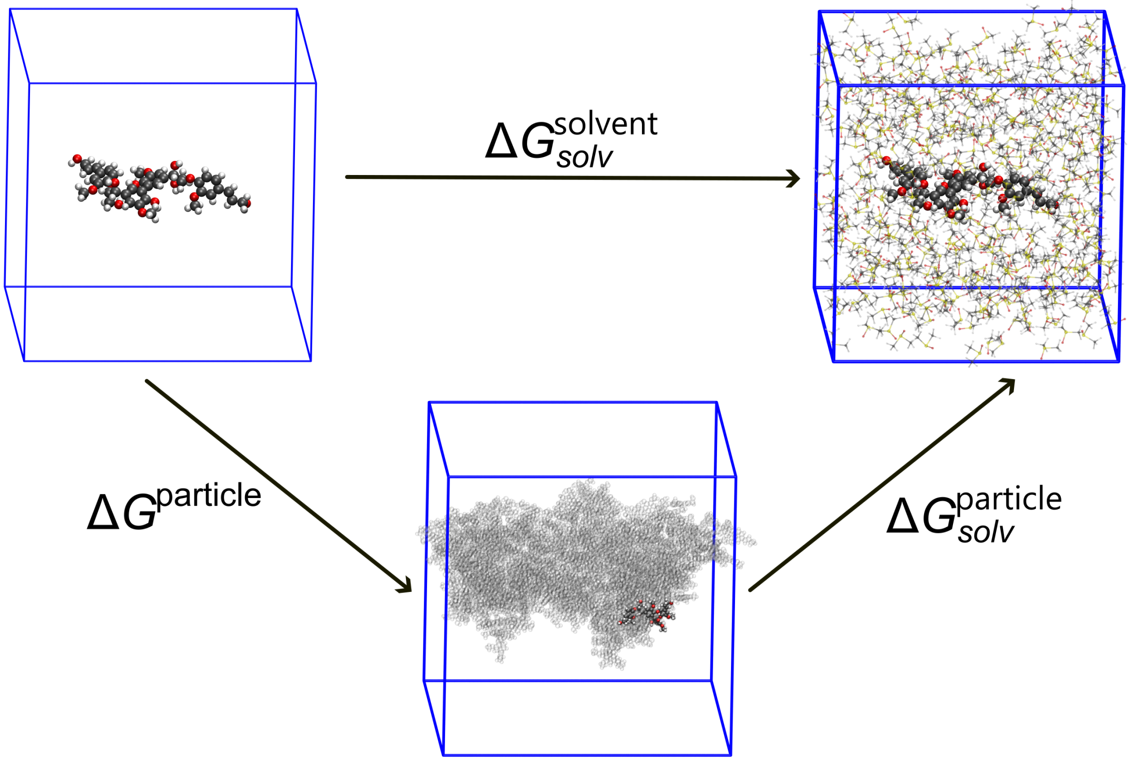

Figure S17: Graphical representation of thermodynamic cycle used to calculate of transfer free energy $\Delta G^{\text{particle}}_{solv}$ from decoupling the lignin molecule from the solid particle $\Delta G^{\text{particle}}$ and solvent $\Delta G^{\text{solvent}}_{solv}$.

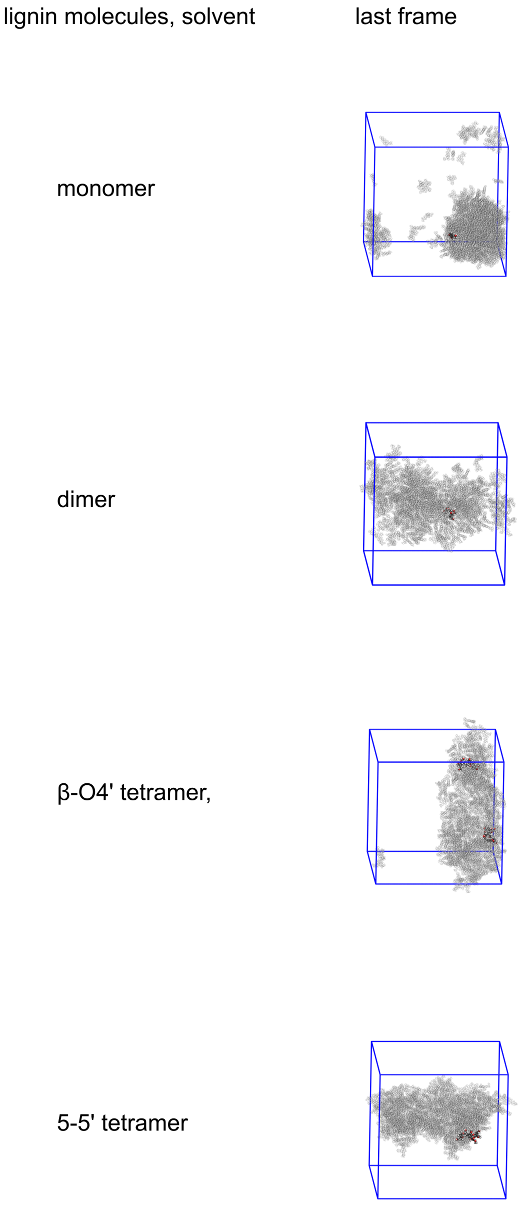

Figure S18: Snapshots of first frame of the simulations of the solid lignin particle showing all lignin molecules as transparent spheres and the decoupled molecule in gray.

Soluble fraction of 5-5' tetramer

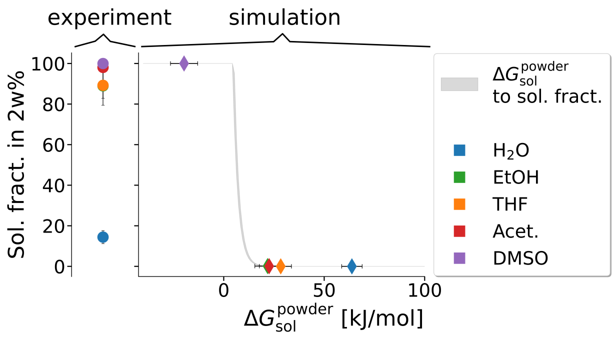

Figure S19: Results of soluble fraction in 2 wt% solution of 5-5' tetramer. Left: experimentally measured soluble fractions. Right: soluble fractions based on calculations of $\Delta G^{\mathrm{aq}}_{solv}$. Gray: Correlation of G^{aq}_{solv} values with soluble fraction showing the exponential relationship.

References

(1) Vermaas, J. V.; Crowley, M. F.; Beckham, G. T. Molecular Lignin Solubility and Structure in Organic Solvents. *ACS Sustainable Chem. Eng.* **2020**, *8*, 17839–17850.
